# Effectiveness of a multilevel intervention to improve mental health of hospital workers: The SEEGEN multicenter cluster randomized controlled trial

**DOI:** 10.1371/journal.pone.0330490

**Published:** 2025-08-21

**Authors:** Nadine Mulfinger, Marc N. Jarczok, Andreas Müller, Melanie Genrich-Hasken, Britta Worringer, Janna Katharina Küllenberg, Florian Junne, Felicitas Rapp, Monika A. Rieger, Eva Rothermund-Nassir, Ute Ziegenhain, Nicole R. Hander, Imad Maatouk, Madeleine Helaß, Martin Peters, Anja Sander, Regina Krisam, Ronald Limprecht, Elena Gesang, Sascha A. Ruhle, Stefan Süß, Bernd Puschner, Peter Angerer, Harald Gündel

**Affiliations:** 1 Department of Psychosomatic Medicine and Psychotherapy, Ulm University Medical Center, Ulm, Germany; 2 Department of Psychiatry II, Ulm University and BKH Günzburg, Günzburg, Germany; 3 Institute of Psychology, Work and Organizational Psychology, University of Duisburg-Essen, Duisburg-Essen, Germany; 4 Institute of Occupational, Social and Environmental Medicine, Centre for Health and Society, Faculty of Medicine, Heinrich-Heine-University Düsseldorf, Düsseldorf, Germany; 5 Institute for Medical Psychology, University Hospital Heidelberg, Heidelberg, Germany; 6 Department of Psychosomatic Medicine and Psychotherapy, University Hospital Magdeburg, Magdeburg, Germany; 7 Department of Anaesthesiology and Intensive Care, Ulm University Medical Center, Ulm, Germany; 8 Institute for Occupational and Social Medicine and Health Services Research, University Hospital Tübingen, Tübingen, Germany; 9 Clinic of Child and Adolescent Psychiatry/Psychotherapy, Ulm University Medical Center, Ulm, Germany; 10 Department of Psychosomatic Medicine, University Hospital Würzburg, Würzburg, Germany; 11 Department for General Internal Medicine and Psychosomatics, University Hospital Heidelberg, Heidelberg, Germany; 12 Institute of Medical Biometry, University of Heidelberg, Heidelberg, Germany; 13 Chair of Business Administration, in particular Work, Human Resource Management and Organization Studies, Heinrich-Heine-University Düsseldorf, Düsseldorf, Germany; 14 Department of Human Resource Studies, Tilburg School of Social and Behavioral Sciences, Tilburg University, Tilburg, the Netherlands; Charite Universitatsmedizin Berlin, GERMANY

## Abstract

**Introduction:**

Hospital workers are at high risk for stress-related mental health issues and are considered a vulnerable workforce in most Western countries. Although multilevel interventions that address individual and organizational factors show promise, there is limited robust evidence of their effectiveness in hospital settings. This study evaluated the SEEGEN trial, a cluster-randomized controlled trial conducted in the German healthcare sector, to assess the effectiveness of a structured multilevel intervention designed to reduce psychosocial stress and to promote mental well-being among hospital employees. The intervention included five modules that targeted different hierarchical levels, sources of interpersonal and structural stress, and potentially vulnerable life stages. These modules were: (i) top management training, (ii) dilemma management – coping by taking responsibility, (iii) promoting stress-preventive relational leadership competence, (iv) reconciling work and family life, and (v) staying healthy at work.

**Methods:**

The study was conducted at three clinical centers in Germany and included 18 clusters with a total of N = 415 participants. The clusters were randomly assigned to either an intervention or a wait-list control group. The primary outcome was psychological strain (Irritation Scale; IRR), and the secondary outcomes were mental well-being (WHO-5) and perceived psychosocial safety climate, (PSC-12). Intervention effects were estimated using a two-level linear analysis of covariance. Changes from baseline to the 11-month follow-up were analyzed.

**Results:**

The intervention had no statistically significant effect on the primary or secondary outcomes.

**Conclusions:**

The lack of significant effects may be attributed to low participation rates, an insufficient intervention dosage, and contextual factors, such as the SARS-CoV-2 pandemic and staffing shortages in the participating hospitals. Although the intervention cannot currently be recommended for widespread implementation, the study provides valuable insights into developing, delivering, and overcoming the challenges of multilevel workplace interventions in healthcare settings.

## Introduction

Several studies have identified psychosocial risk factors as a major source of stress for hospital workers [1–3] and other professions [4–6]. A recent comprehensive review by Rugulies et al. [5] emphasizes the growing burden of work-related mental health issues across various occupations and discusses evidence-based interventions for preventing and managing these issues. While not limited to hospital settings, the review underscores the importance of organizational-level strategies for improving mental health in the workplace. A recent European study [6] quantified the burden of mental disorders and cardiovascular disease attributable to psychosocial work factors, highlighting the importance of improving the work environment at the population level. The Sixth European Survey on Working Conditions [7] showed that, compared to other professions, healthcare workers report not only the most frequent interruptions and the highest work intensity, but also high emotional demands [2]. There is a growing global interest in promoting wellbeing, resilience and self-care among healthcare workers [8].

Although there are some interventions to improve the mental health of healthcare workers, they are usually either targeted at specific occupational groups [9,10] or do not combine individual and organizational interventions [11,12]. However, recent systematic reviews offer a more nuanced view. Aust et al. [13] conducted an umbrella review of 52 systematic reviews, which covered 957 primary studies across sectors. They found moderate to strong evidence that organization interventions, especially those targeting work-time arrangements and task restructuring, can reduce burnout and improve the psychosocial work environment. In a follow-up review, the same group [14] analyzed 22 studies focusing specifically on healthcare workers and showed that nearly 70% of the studies reported improvements in outcomes such as stress, burnout, and mental well-being. Interventions targeting task reorganization and flexible scheduling were particularly effective, especially in reducing burnout among nurses and physicians.

Miguel and colleagues [15] conducted a systematic umbrella review, commissioned by the WHO, which synthesized 16 meta-analyses on workplace mental health interventions. The primary target population was healthcare workers. The interventions were categorized as universal (for all employees), selective (for those at risk), or indicated (for individuals with symptoms). The review primarily focused on psychosocial approaches and found small to moderate improvements in stress, burnout, and general mental well-being. However, using GRADE criteria, the overall certainty of evidence was rated as low to very low. The authors emphasized the importance of tailoring interventions to the specific needs of employees and the contexts of their workplaces, arguing that individualized approaches are more likely to produce sustainable improvements in mental health outcomes.

In contrast to individual interventions aimed at improving the well-being of employees, organizational interventions aimed at addressing the causes of occupational stress have been less researched [16]. In their systematic review of workplace interventions to address well-being and burnout in hospital workers, Cohen and colleagues [12] found that only three of the 33 included studies were organizational interventions. This indicates a clear underrepresentation of structural approaches. Furthermore, the authors caution against overinterpreting the results due to limited rigor and methodological heterogeneity of the included studies. These limitations underscore the necessity of more rigorous evaluations of organizational-level strategies in healthcare settings. However, organizational interventions may be more effective for employee well-being [12]. In a systematic review of the impact of organizational interventions on physician burnout, DeChant et al. [17] showed that workplace interventions that improve workflow, optimize electronic health records, reduce administrative burden through the use of clerical staff, and introduce team-based care can indeed reduce physician burnout. There is also convincing evidence that interventions that address both individual and organizational aspects are particularly effective in preventing physician burnout [18]. Other reviews found that effectiveness varied widely [9], with better effects for organizational interventions [19].

In addition, involving staff in the development and implementation of an intervention can be a catalyst for change [20]. Results from a longitudinal study of the implementation of teamwork in Denmark show that participation and changes in work procedures were significantly associated with autonomy, social support and well-being after the intervention [21]. However, Montano et al. [22] recently reported that their participatory organizational intervention study had no impact on the working conditions of the majority of healthcare workers (about 70%). The authors describe the main shortcoming of the intervention as the lack of an evidence-based framework linking specific interventions to expected outcomes.

Based on this specific research, our consortium aimed to develop a set of interventions for different healthcare professionals in different types of clinics: Individual interventions were to focus on key issues identified through needs assessment in hospitals, through interaction with the target groups, and based on theoretical and practical work, and tested in pilot studies, such as leadership, as well as life-span issues (work-family issues, ageing). The theoretical underpinnings of each of these intervention modules are explained in the methods section.

Within this framework, Irritation (IRR) was selected as the primary outcome of the study. This concept captures the subjective perception of emotional and cognitive stress in response to work-related demands, and it is theoretically grounded in Lazarus’s transactional stress model [23,24]. IRR is considered a key indicator of psychological strain in occupational contexts because it represents a transitional psychological state between mental fatigue and clinical symptoms [25].

The concept of psychosocial safety climate (PSC) attributed to the theoretical background of our multilevel intervention. The concept is concerned with workplace support at the team and organizational levels [26]. The PSC includes “[...] policies, practices, and procedures for the protection of worker psychological health and safety” and also the level of support and approval from those within the organization [27; p.580]. Dollard and Bakker [27] identified the PSC both as a predictor of change in individual mental health problems through its relationship with individual job demands and as a moderator between emotional demands and emotional exhaustion among employees of the Australian Department of Education.

Against this background, this multi-site cluster randomized controlled trial (cRCT) tested the effectiveness of a new multilevel intervention combining individual and organizational aspects. This manuscript aims to evaluate the effectiveness of this multilevel intervention in improving psychosocial outcomes among healthcare workers. The specific objectives are: (1) assessing changes in psychological strain (measured by IRR) as the primary outcome, and (2) exploring the effects of the intervention on secondary outcomes, including mental well-being (measured by the WHO-5) and perceived psychosocial safety climate (measured by the PSC-12). We hypothesize that, at the 11-month follow-up, participants in the intervention group will show significantly greater improvements in these outcomes compared to the control group.

## Materials and methods

### Study design

The study used a cluster-randomized, multicenter design with three clinical centers: A) a hospital owned by a private healthcare company, B) a community hospital, and C) a university hospital. Organizational units (e.g., departments or wards) within each site served as clusters and were randomly assigned to the intervention or control condition (see below for details on randomization).

Data collection took place between December 2019 and January 2021. Individual participant data (sociodemographic and individual-level questionnaires) were collected simultaneously at each study site: baseline (T0 between 01 December 2019 and 31 January 2020), mid-intervention (T1 between 01 June and 31 July 2020), and post-intervention (T2 between 01 December 2020 and 31 January 2021), either on paper in case report forms or web-based.

Data management and analysis were performed by the Institute of Medical Biometry (IMBI, University Hospital of Heidelberg) according to the statistical analysis plan (SAP). Results are reported according to the CONSORT guidelines for cluster randomized trials [28, S1 Table]. The full study protocol was published prior to data collection in phase II [29].

### Participants and procedure

Inclusion criteria were employment in patient care at one of the three study sites, age 18–70 years, sufficient knowledge of German to complete the questionnaires, and written informed consent. Information sessions and workplace health management at the three sites helped to inform interested employees about the study and the different interventions. In addition, potential study participants were approached directly by project staff in all wards of the participating clinics. A combination of department-level presentations by project staff and individual support from the company’s health management team was essential to achieving a significant portion of the targeted sample size. This support primarily consisted of promoting the sessions and informing staff through internal channels.

**Fig 1:**
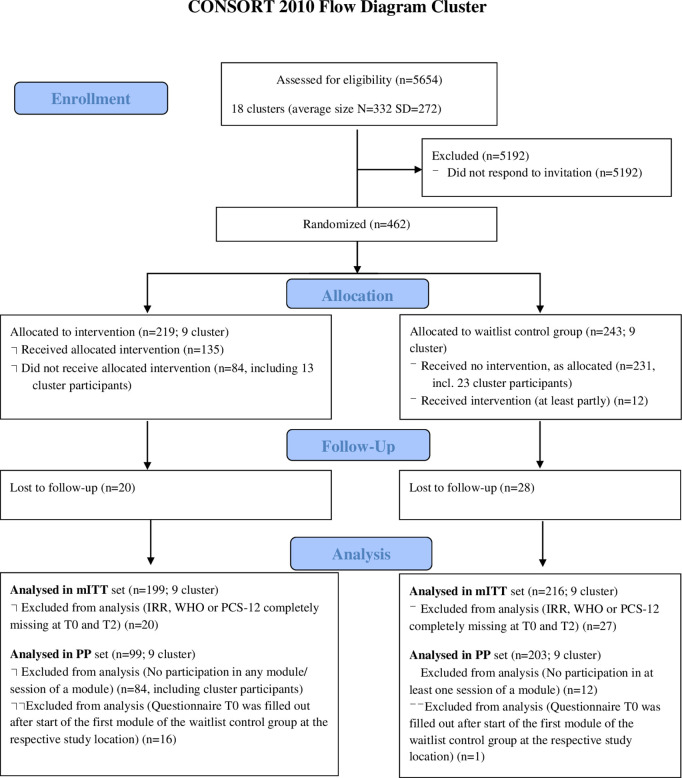
CONSORT flow diagram. mITT: modified intention-to-treat; PP: per protocol.

**Fig 2:**
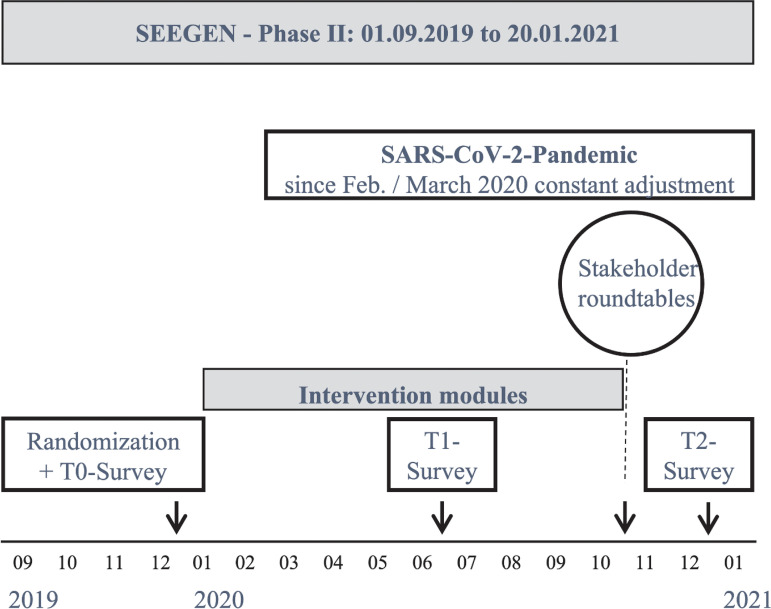
Timeline of SEEGEN phase II.

The three study sites were recruited through existing SEEGEN network collaborations. Participation was voluntary and required formal approval from the senior management of each hospital. Prior to inclusion, discussions were held with key administrative and occupational health representatives to ensure organizational support and feasibility. As part of these agreements, it was explicitly defined that up to 12 hours of regular working time could be used by staff to attend the intervention modules. Due to differences in length of models, a module participation was defined as a minimum of 50% of attendance in the module. All sites initially signalled strong interest in supporting workplace mental health and agreed to implement the intervention within the defined framework. A total sample size of 18 clusters (six per site, multiple departments per cluster) with a 1:1 allocation ratio for the intervention group (IG) and waiting list control group (WCG) was assessed. In addition, staff who did not participate in any of the modules but who wished to participate in the SEEGEN study by answering shorter questionnaires (cluster participants) were included to measure the diffusion effects of the SEEGEN study. For more details, see Figs 1 and 2.

Institutional review board approval was obtained at each study site (IRB 1: Heinrich-Heine-University Düsseldorf: Az: 6193R; IRB 2: University of Heidelberg: Az: S-602/2019; Ulm University: Az: 501/18). This trial was conducted in accordance with the tenets of the Declaration of Helsinki. All participants provided written informed consent. The SEEGEN trial was registered in the German Clinical Trials Register (DRKS) under the DRKS-ID DRKS00017249 (8 October 2019) https://drks.de/search/en/trial/DRKS00017249.

### Deviations from the registered and published protocols

While the trial was largely conducted in accordance with the registered and published protocols, several deviations occurred due to practical and contextual constraints, particularly those related to the SARS-CoV-2 pandemic:

- Sample size: The final sample size was smaller than initially planned due to challenges with recruitment and retention (see below).
- Follow-up (T2): Originally scheduled nine months after baseline (T0), the follow-up was postponed to 11 months after T0 to account for pandemic-related delays and site logistics.
- Mode of intervention delivery: Due to infection control measures during the SARS-CoV-2 pandemic, several seminars originally planned as in-person events were conducted online to ensure continuity of the intervention.
- Primary outcome assessment: The original protocol planned to assess the primary outcome (IRR) from T0 to T1. This was revised to assess change between T0 and T2 to better capture the intervention’s sustained effects.
- Intervention duration: The intervention period was extended into 2021 (instead of ending in 2019 as initially planned) following a formal amendment and ethics approval.
- Booster sessions: The two-hour booster sessions originally planned to follow each intervention module were not conducted as such. Instead, a single, joint booster session (or roundtable) was held after the completion of all modules with all intervention participants. The issues and ideas gathered during these sessions were then presented at a subsequent stakeholder roundtable.

### Intervention

**Fig 3:**
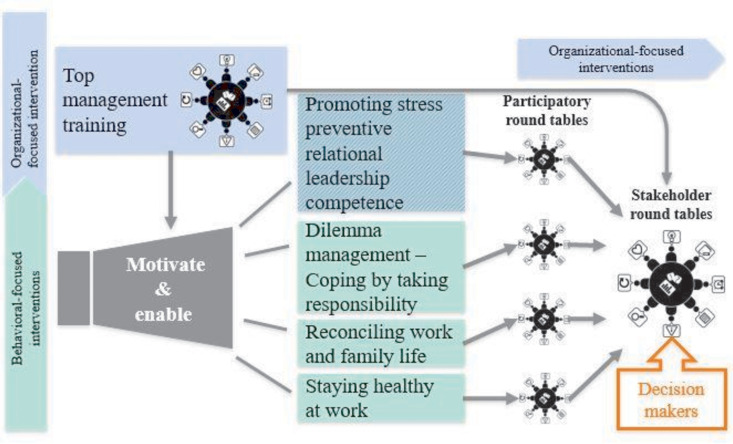
Structural model multilevel intervention.

The elements of this multilevel intervention emerged from the collaboration within the SEEGEN (Mental health in the workplace hospital, German: Seelische Gesundheit am Arbeitsplatz Krankenhaus) consortium and were developed in two phases: Phase I identified the most vulnerable target groups and developed specific intervention modules to address A) the needs of the hospital workers, B) the different hierarchical levels within hospitals and C) multiple functional areas. The feasibility and the effectiveness of the developed modules were also evaluated during this phase and have been previously published [30–36]. In phase II, a unique, multilevel intervention was implemented and tested in three German hospitals using a cRCT. This intervention combined previously tailored modules with complementary organizational interventions, such as participatory roundtables. The intervention was conceptually informed by the IGLO framework developed by Nielsen & Christensen [37], which emphasizes the need for coordinated action at the individual, group, leadership, and organizational levels to promote sustainable changes in mental health and work participation. This multilevel structure guided the development and implementation of five modules, which included individual and organizational participatory approaches. These approaches were piloted before implementation (see Fig 3).

The SEEGEN multilevel intervention consisted of the following five modules: (i) top management training, (ii) dilemma management – coping by taking responsibility, (iii) promoting stress-preventive relational leadership competence, (iv) reconciling work and family life, and (v) staying healthy at work. Our consortium developed a series of interventions for different healthcare professionals in different types of clinics: Distinct interventions were designed to address identified key issues, such as leadership training, as well as issues embedded in the life course (e.g., work-family issues). These interventions were designed to provide a safe space to reflect on important personal issues and to explore individual solutions. Recurrent individual issues raised by staff in these seminars, which reflected problems within the organization, were collected and then addressed in a final series of organizationally focused interventions (‘roundtables’). In phase I of the SEEGEN study, each intervention module was adapted to the specific needs of the hospital staff. Before and during Phase I of the SEEGEN study, various intervention modules were offered and evaluated in five pilot projects [30–36], each addressing a specific target group of hospital staff (e.g., nurses). The promising results of these evaluations informed the design of the current trial.

For example, the Staying healthy at work intervention was found to be effective for nurses aged 45 and older regarding mental health-related outcomes, based on a multicenter randomized controlled trial [30]. Similarly, the Reconciling work and family life intervention was also found to be effective: Compared to the control group, the IG showed a significant increase in self-efficacy was shown in before and after the intervention. Furthermore, most participants reported high satisfaction. Group exchange and therapeutic input were especially regarded as relevant [31].

Dilemma management training was piloted and evaluated as a 10-month training program, administered to N = 69 senior physicians, senior nurses and senior service and administrative staff in four hospitals. Overall, participants showed less cognitive irritation and less perceived stress reactivity over time [32]. Qualitatively, coping responses changed over the training period [33]: Before the training program began, most trial participants exhibited impulsive (e.g., eruptive and highly emotional) and automatic (e.g., standard) strategies in dilemma situations. These strategies included desperately trying to resolve the situation by satisfying all demands simultaneously or postponing urgent decisions. After the training period, most participants used strategies that they consciously selected from a range of alternatives. These strategies included reflecting on conflicting demands and comparing pros and cons before making a justifiable decision.

The stress preventive leadership intervention for middle management was piloted with N = 93 leaders of middle management in a tertiary hospital [34]. Participants evaluated the intervention as highly acceptable and feasible. Quantitative data analysis showed intraindividual improvements in cognitive irritation [38], well-being [39] and transformational leadership [40] after participation [35]. Furthermore, qualitative data analysis revealed that leaders perceived improvements in their daily leadership behavior, such as increased awareness of appreciation, reflection on their leadership roles, and prioritization of employee concerns. Additionally, they became aware of their limitations due to their middle management position [36].

The design and development of the intervention modules used in this trial were informed by these findings from Phase I and earlier. The hospitals involved in Phase I of the SEEGEN study were different from the sites included in the current trial. The intervention modules were delivered in a group setting. The intervention modules were delivered A) across different service levels, B) across different hierarchical levels within each hospital, and C) across functional areas to capture relevant dimensions of the hospital environment. Participants were invited to choose a module according to their specific needs and interests. The maximum duration of each module in phase II was limited to 12 hours, with a minimum of six hours. Staff could attend modules during normal working hours and allocate their time budget to either one 12-hour module (ii, iii, or v) or two six-hour modules (i and iv). The modules were delivered by multidisciplinary teams of experienced trainers. These teams consisted of a mix of research group members and external consultants who were not involved in the study design or data collection. In order to be considered as having participated in the intervention, individuals had to attend at least one full day of a two-day module, or at least half a day of a one-day module.

(i) Top management training

In the overall concept, this sub-project represents a kick-off workshop aimed at clinical leaders in medicine or nursing. The focus of the workshop is to sensitize senior nurses and senior physicians to the issue of healthy work design, and to prepare the ground for the other approaches of the multilevel intervention. The module aimed to introduce participants to a 4-step plan for implementing preventive measures. The first step is to discuss options for conducting a psychosocial risk assessment, the second step is to discuss verbalising and prioritising change goals, the third step is to develop an action plan, and the final step is to develop options for evaluation and performance review.

(ii) Dilemma management—Coping by taking responsibility

The aim of this two-day training is to equip healthcare workers with the mental and behavioral skills to manage conflicting demands in their organization, to regain control in dilemma situations and to act constructively in decision-making processes. The training aimed to raise participants’ awareness of organizational paradoxes and interactible dilemmas. Participants were encouraged to reflect on their own cognitive, emotional, and behavioral response patterns. The trainer guided them through this process using structured prompts and group discussions.

(iii) Promoting stress-preventive relational leadership competence

This module targeted the stress prevention role of middle managers by focusing on leaders’ own stress coping skills and healthy leadership style, particularly transformational leadership (e.g., [41]). The first session was designed to help leaders’ cope with work-related stressors, while sessions two, three and four were planned to address leadership style [40,42] and mindful communication as well as leading teams by anticipating team dynamics.

(iv) Reconciling work and family life

This module was proposed for parents with children aged 0–18 years. The training was specifically designed to promote awareness of the individual work-family conflict, to identify and strengthen individual resources, and to empower individuals to practice appropriate self-management techniques by combining behavioral therapy techniques, emotion-centered principles and group dynamics. The module included a planned practical session on stress management, to be facilitated by an experienced yoga instructor.

(v) Staying healthy at work

In six modules spread over 1.5 days, the participants discussed practical exercises for everyday life. The trainers were instructed to encourage interaction between the participants. The central theme of the first day was the “Theory of Selection, Optimisation and Compensation (SOC)” [43]. This approach involves a resource-oriented focus and an optimal use of resources despite increasing age-related limitations. On the second day, the focus was on developing individual SOC projects. For instance, one nurse chose to reduce fatigue during night shifts. She optimized this goal by coordinating adjusted shift blocks with her colleagues, and compensating for remaining fatigue with brief relaxation exercises and short naps during breaks. These individual projects were developed and refined throughout the seven weekly intervention sessions as outlined by Maatouk et al. [30].

In addition to the individual-level intervention modules, two interventions, the “top management training” and the “promoting stress preventive relational leadership competence”, as well as various stakeholder roundtables, were held to facilitate structural and organizational changes and to develop organization-specific proposals for action. Two types of roundtables were held at each of the three intervention sites. First, a roundtable was held at each site to synthesize feedback from all module types and formulate initial proposals for organizational improvements. Second, a consecutive stakeholder roundtable was held at each site to discuss and refine these proposals prior to implementation. These stakeholder roundtables included key decision-makers from hospitals, as well as selected participants from previous module roundtable discussions. This ensured that improvement suggestions were developed collaboratively and in a participatory manner. Concrete proposals for organizational improvements were developed across the roundtables. These proposals addressed measures on topics such as information and communication flow, leadership and respectful interaction, onboarding of new staff (including intercultural orientation), staff retention, discharge and bed management, installing short-term care beds, staff break regulations, hospital-based childcare, and staff training. Approximately eleven proposals covering five to six thematic areas were formulated, with multiple concrete suggestions per topic. In addition to the co-created proposals, the roundtables also included structured presentations and collaborative methods, such as the World Café, to promote knowledge exchange between different hierarchical levels [44]. Roundtables were scheduled to last 2–4 hours each. Stakeholder roundtables serve as a participatory approach within the hospital to select, address and find the best possible solutions to a hospital’s most salient problems identified during the intervention phase and by means of analyzing staff feedback after the seminars.

### Outcomes

The primary outcome was measured using the Irritation Scale [IRR, 38]. The IRR consists of eight items each rated on a 7-point Likert scale ranging from 1 (=not at all true) to 7 (=almost completely true). The total score ranges from 8 to 56. The change in IRR from T0 to T2 is defined as: IRR_T0_T2* = IRR_T0 – IRR_T2* ranging from −48–48, where a positive value indicates an improvement, i.e., less irritation between T0 and T2. The scale has been widely used to monitor organizational stress and evaluate preventive interventions [38].

Secondary outcomes included well-being and psychosocial safety climate. Well-being was measured using the five-item World Health Organisation Well-Being Index [WHO-5; 39]. The five items are each rated on a 6-point Likert scale ranging from 0 (=never) to 5 (=all the time). The total score of the WHO-5 is the sum of the five items multiplied by 4 and ranges from 0 (= the worst well-being) to 100 (=best well-being). The change in the WHO-5 sum score ranges from −100 to 100, with a positive value indicating an improvement in well-being between T0 and T2.

The Psychosocial Safety Climate Scale [PSC-12; 45] consists of four dimensions: (1) organization participation (3 items), (2) organization communication (3 items), (3) management priority (3 items) and (4) management commitment (3 items). Each item is rated on a 5-point Likert scale from 1 (=strongly disagree) to 5 (=strongly agree). The total PSC-12 score is the sum of the 12 items ranging from 12 to 60. The higher the PSC-12 score, the better the psychological safety climate. According to the PSC benchmark risk level [46] scores ≤ 26 are associated with very high risk of impaired mental health (very low PSC; high risk PSC 37 ≤ and ≥ 27).The change in the PSC-12 ranges from −48 to 48, with a positive value indicating an improvement in the psychological safety climate between T0 and T2.

### Sample size

The sample size calculation is based on the primary endpoint, i.e., the difference between IG and WCG in the absolute change in the total IRR score before and after the intervention, assessed by an ANCOVA (see statistical analysis). The initial sample size was calculated for a medium effect (d = 0.4, two-sided α = 0.05, power 1-β = 0.8). Including attrition and design effects, a final sample size of 720 was calculated for 18 clusters (six per study site, see 29). The sample size is based on the absolute change in the total IRR score from T0 to T2*, which is evaluated using an ANCOVA approach. A two-sided, two-sample t-test with a significance level of α = 0.05 and a power of 1-β = 0.8 was used to provide a conservative estimate of the sample size required for the ANCOVA. Assuming that the intervention has a medium effect (d = 0.4), this results in a required sample size of 100 participants per group (200 total for intervention and control). To account for clustering effects and to be conservative, an intraclass correlation coefficient (ICC) of 0.05 and an average cluster size of 40 participants were assumed, resulting in a design effect of 1 + 0.05 × (40–1) = 2.95. This results in a total required sample size of 2.95 × 200 = 590 participants. Considering an expected dropout rate of 18%, the total sample size increases to 720 participants across 18 clusters. To account for potential cluster dropouts, more than 18 clusters may be recruited, which would increase statistical power. Sample size was calculated using SAS 9.4.

### Randomization

Cluster randomization was stratified by the three sites in a 1:1 ratio prior to recruitment of the first participants in phase II. Cluster pairs per site were defined and randomized with a block size of two to ensure equal distribution of clusters at each site. Randomization was performed by the IMBI independently of the other study personnel in a concealed manner. Cluster pairs per site were predefined in order to prevent matched pairs from being randomized to the same group. Within each site, pairs of organizational units (i.e., wards) were defined based on their structural similarity. This is an important step to avoid both, comparing, e.g., a small service unit with a large surgery unit, as subunit-specific organizational needs can vary greatly. It also helps to avoid spillover effects between intervention organisation unit and control organization units that share many facilities and personnel. Therefore, similar pairs were carefully selected and randomly assigned to the intervention or control group. The amount of clustering within the clusters per hospital is measured by calculating the intra-class correlation coefficient for the difference between the baseline value of the total score of the IRR at T0 and the total score of the IRR at T2 by using a linear two-level ANCOVA model only including the random intercept for cluster. We used a validated SAS macro with a fixed and pre-specified seed for random number generation to ensure reproducibility. Participants and researchers were not blinded to the intervention aims or group allocation. However, the study statisticians were blinded.

### Statistical analysis

Descriptive analyses of differences between groups are expressed as mean average values with corresponding standard deviations (SDs) or as frequencies and corresponding percentages. The primary and secondary outcomes were analyzed using linear two-level linear analysis of covariance (ANCOVA) models. These models tested the null hypothesis that the absolute change between T0 and T2 in the total score of the primary (IRR) and secondary outcomes (WHO-5, PSC-12) in the IG is equal to the absolute change in the total score in the WCG. Models included the baseline value of the total score for each outcome (i.e., T0). Covariates were the hierarchical level (top management vs. middle management vs. staff with no management responsibility), participant gender (female vs. male vs. diverse and missing data), and study site and cluster as random effects. The significance level was set at *α* = 5% (two-tailed). The hierarchy level of the current position was included as a covariate to account for systematic differences between organizational roles, such as decision-making responsibility, influence, and access to resources. These aspects are relevant both for how participants engage with the intervention and how they may respond to the intervention. The intervention modules were also tailored to these levels, with distinct content developed for top managers, middle managers and staff without leadership roles. Including this variable reduced potential confounding factors and allowed for a more accurate estimation of intervention effects on heterogeneous target groups.

To account for structural differences between sites, hospital type was included as a covariate in the analysis. These differences may reflect variations in organizational structure, available resources, staffing levels, and institutional culture – all of which could plausibly affect the implementation and reception of the intervention.

The degree of clustering within the clusters per hospital was measured by calculating the intra-class correlation coefficient for the difference between the baseline value of the total IRR score at T0 and the total IRR score at T2 by using a linear two-level linear ANCOVA model including only the random intercept for cluster (IRR 0.0271 ± 0.0124 from N = 10 imputations). This hierarchical testing strategy was used to control for the overall type I error. The primary analysis was performed on a mITT set, excluding participants with missing IRR, WHO-5 or PSC-12 scores at both T0 and T2, so that the primary and key secondary endpoints could not be calculated [29]. Although the outcome measures are ordinal in nature, they were treated as approximately interval-scaled, as is common in comparable intervention trials. This approach permitted the use of linear ANCOVA models, which allowed for adjustments to be made for multiple covariates and hierarchical clustering.

The per-protocol (PP) set was analyzed as a sensitivity analysis. The PP set was a subset of the mITT set and included all study participants who participated according to the study protocol. Participants were excluded if they (i) were randomized to the IG, but did not receive any intervention session in any module; (ii) were randomized to the WCG, but received at least one intervention session in any module; (iii) completed the T0 assessment after the first invention module was started at each study site (i.e., no baseline); (iv) or completed the T2 questionnaire after the start of the first module of the WCG at each study site.

According to the study protocol [29] the primary outcome was originally between T0-T1. Corona-related adjustments, i.e., delays, resulted in the operationalisation of the change from T0 to T2 (Fig 2).

Missing values were imputed using multiple imputation for the primary analysis of the primary endpoint and key secondary endpoints. Missing values were imputed on single item level using the predictive mean matching method. Imputation by fully conditional specification (FCS) was used, which provides a flexible method to specify the multivariate imputation model for arbitrary missing patterns including both categorical and continuous variables, see also 29 (2019). The imputation model for the primary and secondary endpoint consists of all variables used for the respective analysis: the respective outcome items at T0 and T2, gender, hierarchy level, treatment group and hospital site. Imputation rates were as follows: IRR = 37.5%, WHO-5 = 39.3%, PSC-12 = 38.1%. These imputation rates represent the proportion of missing values that were replaced by multiple imputation. Although the proportion of missing data was relatively high overall, the methodological approach to handling missing data – including the use of multiple imputation and complete-case sensitivity analyses – was based on published recommendations for clinical trials and cluster-randomized trials [47,48]. These sources support the use of multiple imputation under the missing-at-random (MAR) assumption, even in studies with small sample size. They also emphasize transparent reporting in accordance with CONSORT guidelines [28]. To ensure the reliability of our findings, we performed additional sensitivity analyses based on complete cases, yielding comparable results to those of the imputed analyses.

### Data handling and handling of missing values

Invalid questionnaires or single items (e.g., multiple answers instead of one) were present in <1% of participants in IRR and WHO-5 at both, T0 and T2, but in 6% of mITT participants in PSC-12 T0 and T2. These responses were set to missing. Missing values were imputed using multiple imputation for the primary analysis of the primary endpoint and key secondary endpoints. Missing values were imputed at item level using the predictive mean matching method with fixed seed for reproducibility (m = 10 imputations). As the items were measured at an ordinal level with a relatively high number of categories (IRR: categories 1–7; WHO-5: categories 0–5; PSC-12: categories 1–5), the predictive mean matching method was chosen. Imputation by fully conditional specification was used. The imputation model for the primary and secondary endpoints included all variables used in the analyses: all IRR (or WHO-5/PSC-12) items at T0 and T2, gender, hierarchy level, treatment group and hospital site. As there were no convergence problems and the extended imputation models also converged, all corresponding items at T1 were additionally included in the respective models. For the multiple imputation of hierarchy level, the logistic regression model was defined in the statistical analysis plan (SAP). However, as the hierarchy level is nominal but not ordinal, the discriminant method was used instead. Gender, allocation and hospital site were used for the imputation of hierarchy level. In addition, the total IRR, WHO-5 and PSC-12 scores at T0, T1 and T2 were included to ensure equivalence of imputed and observed values for the primary and main secondary endpoints. As only one participant reported diverse as gender, gender was not imputed due to expected convergence problems. As convergence problems were also expected in the imputation models for IRR, WHO-5, PSC-12 items and hierarchy level, the gender ‘diverse’ and missing values were collapsed into one category. To minimize uncertainty, ten imputed datasets were generated for the analysis of the primary and main secondary endpoints. The results from each of these were combined (mean effects, standard errors using Rubin’s rules). Rubin’s rules are a set of formulas that combine estimates (e.g., means and standard errors) from multiple imputed datasets to produce a single, valid overall result. This approach considers both within- and between-imputation variability. Missing data for the mITT and the PP sets were imputed separately. For imputation diagnostics, the distribution of the observed and imputed data was checked descriptively using histograms.

In the absence knowledge of the results, the following definition of participant leadership was used: Hierarchy level reported as ‘top management’ or ‘middle management’ was categorized as leadership responsibility. In case of missing information on hierarchy level, the variable managerial position is considered and counted as leadership responsibility if the answer is ‘yes’. There were no participants with missing hierarchy level and managerial position. Staff responsibility (i.e., holding a leadership or supervisory position) was not included as a covariate and was therefore not statistically controlled for in the analysis.

## Results

A total of N = 5.654 workers were eligible for inclusion. N = 462 participants from 18 clusters gave written informed consent (8.2%), with a total of n = 219 in IG and n = 243 in WCG. The response rates were as follows: T0: 87.88%; T1: 68.40%; T2: 46.65%. A total of 47 participants were excluded due to missing information on the primary or secondary outcomes (n = 20 from IG, n = 27 from WCG, Fig 1). Baseline characteristics of participants were reported descriptively by group. In accordance with current recommendations for randomized trials, no statistical testing was conducted on baseline variables [49,50]. The modified intention-to-treat (mITT) set consists of 415 participants (N = 199 in IG; N = 216 in WCG), the per protocol (PP) set of 302 participants (N = 99 in IG; 203 in N = WCG). See Fig 2 for the timeline of SEEGEN in phase II.

Table 1 shows descriptive statistics for the participants (N = 415). Participants from the IG were less likely to be in a managerial position, less likely to be in middle management or above and less likely to have managerial responsibilities. There were also no differences between IG and WCG in terms of total IRR, WHO-5 and PSC-12 scores. In accordance with current guidelines and methodological standards, no statistical significance testing was performed on baseline variables because random allocation is expected to ensure group comparability. See Fig 1 for participant flow. Overall, our study participants represent approximately 8% of all employees at the three sites.

**Table 1:** Participant characteristics.

	IG (n = 199)	WCG (n = 216)
Age (years), mean (SD)	44.0 (11.8)	43.0 (11.2)
Gender, n (%)		
Male	52 (27.2)	49 (23.4)
Female	139 (72.8)	159 (76.1)
Diverse	0 (0.0)	1 (0.5)
Marital status, n (%)		
Married/in partnership	135 (71.4)	153 (74.6)
Single	34 (18.0)	40 (19.5)
Divorced	17 (9.0)	9 (4.4)
Widowed	3 (1.6)	3 (1.5)
Leadership position, n (%)	67 (33.7)	96 (44.4)
Leadership responsibility, n (%)	67 (33.7)	99 (45.8)
Current working situation, n (%)		
Full-time	124 (64.9)	137 (66.2)
Part-time	66 (34.6)	68 (32.9)
Parental leave/other leave of absence	0 (0.0)	2 (1.0)
Other	1 (0.5)	0 (0.0)
Shift work n (%)		
No	88 (46.3)	91 (44.2)
Yes	102 (53.7)	115 (55.8)
IRR Total Score, mean (SD)	26.0 (9.5)	26.2 (9.6)
WHO Total Score, mean (SD)	55.1 (18.9)	58.2 (18.3)
PSC-12 Total Score, mean (SD)	28.7 (9.9)	29.5 (9.5)
Hierarchy level of current position n (%)		
Top management,	13 (7.0)	16 (7.8)
Employee without management responsibility	123 (65.8)	109 (53.2)
Middle management	51 (27.3)	80 (39.0)
Working group n (%)		
Medical service	52 (27.7)	57 (28.2)
Medical-technical service	9 (4.8)	7 (3.5)
Nursing service	94 (50.0)	96 (47.5)
Functioning service	23 (12.2)	22 (10.9)
Secretariats	4 (2.1)	4 (2.0)
Other	6 (3.2)	16 (7.9)

Notes: mITT = modified intention to treat; SD = standard deviation; IG = intervention group; WCG = waitlist control group; number of missing not included in %, because of multiple imputation. Missing values (IG/WCG): age (13/13); WHO-5 (6/10). Missing values IG/WCT: age (13/13); gender (8/7); marital status (10/11); current working situation (8/9); shift work (9/10); IRR Total (6/8); WHO Total (6/9); PSC-12 Total (6/12); Hierarchy level of current position (12/11); Working group (11/14); medical service: hospital medical staff responsible for providing inpatient and outpatient care, including physicians.

A total of 217 module visits were observed. They were distributed as follows: Staying healthy at work n = 60, dilemma management n = 57, top management training n = 40, promoting stress-preventive leadership n = 39, and reconciling work and family n = 21.

### Primary and secondary outcomes

Table 2 shows the mean and SD by group and time point, including the effect sizes for the change score between IG and WCG.

**Table 2:** Primary and secondary outcomes at T2 and change between T0-T2.

mITT	T2		Change between T0-T2		Effect size for between group change
	IG (n = 199)	WCG (n = 216)	IG (n = 199)	WCG (n = 216)	Cohen’s d [95%CI]
IRR total score	26.5 (10.0)	26.5 (9.9)	−0.14 (7.1)	−0.98 (7.2)	.12 [−.075 –.311]
WHO total score	52.6 (20.9)	52.1 (21.6)	−2.31 (14.5)	−6.24 (16.6)	.25 [.058 –.444]
PSC-12 total score	28.7 (10.3)	28.9 (9.0)	0.32 (7.08)	−0.70 (7.12)	.10 [−.090 –.295]
**PP**	**T2**		**Change between T0-T2**		**Effect size for between group change**
	**IG (n = 99)**	**WCG (n = 203)**	**IG (n = 99)**	**WCG (n = 203)**	**Cohen’s d [**95%CI]
IRR total score	26.9 (10.6)	26.5 (10.0)	0.11 (7.30)	−1.15 (7.08)	.18 [−.065 –.417]
WHO-5 total score	52.9 (20.4)	51.8 (21.8)	−0.44 (14.6)	−6.17 (16.8)	.35 [.113 –.597]
PSC-12 total score	27.6 (10.2)	29.0 (8.7)	−0.36 (6.82)	−0.74 (7.13)	.05 [−.185 –.295]

Notes: mITT = modified intention to treat; PP = per protocol; IG = Intervention group; WCG = waitlist control group; IRR = Irritation; PSC-12 = psychosocial safety climate. All descriptives are mean (sd). Effect size Cohen’s d for group differences in Change from T0-T2.

Small effect sizes were observed for all three outcomes (Cohen’s d 0.1–0.25). The 95% CI included the zero, indicating a lack of statistical significance for IRR and PSC-12. In both groups, the WHO-5 score decreased from T0 to T2, with the average decrease in well-being being greater in the WCG than in the IG. The effect sizes for IRR change score (d = 0.18) and WHO-5-100 (d = 0.35) increased slightly in the PP analysis. The effect size for the PSC-12 score decreased (d = 0.05). The results for the primary and key secondary outcomes from the ANCOVA models are shown in Table 3.

**Table 3:** Primary and secondary outcomes estimation (ANCOVA).

	mITT (N = 415)	PP (N = 319)
IRR Change T0–T2	Estimate [95%CI]	Estimate [95%CI]
Intercept	**8.87 [4.68–13.06]**	**8.34 [4.20–12.48]**
**IRR Score at T0**	**−0.34 [−0.44 – −0.24]**	**−0.27 [−0.39 – −0.15]**
Group (IG)	−0.51 [−2.49–1.47]	−1.09 [−3.17–0.99]
Group (WCG)	Reference	Reference
Hierarchy level (top management)	1.46 [−2.40–5.32]	1.58 [−2.26–5.42]
Hierarchy level (middle management)	−0.03 [−1.74–1.68]	0.06 [−2.29–2.41]
Hierarchy level (no management)	Reference	Reference
Male	Reference	Reference
Female	0.32 [−1.64–2.28]	−0.09 [−2.48–2.30]
Divers or missing	3.03 [−2.12–8.19]	7.16 [−1.15–15.47]
Hospital owned by a private healthcare company	Reference	Reference
Community Hospital	−0.21 [−2.97–2.55]	−1.28 [−3.85–1.29]
University Hospital	0.39 [−2.37–3.15]	−0.05 [−2.93–2.83]
**WHO-5 Change T0–T2**		
Intercept	**13.93 [5.14–22.71]**	**14.09 [2.74–25.44]**
**WHO-5 (100) at T0**	**−0.33 [−0.43 – −0.23]**	**−0.33 [−0.45 – −0.21]**
Group (IG)	2.63 [−1.80–7.06]	4.02 [−1.23–9.27]
Group (WCG)	Reference	Reference
Hierarchy level (top management)	−3.16 [−11.92–5.60]	−5.7 [−15.25–3.85]
Hierarchy level (middle management)	−0.47 [−4.43–3.49]	0.26 [−4.84–5.36]
Hierarchy level (no management)	Reference	Reference
Male	Reference	Reference
Female	−3.73 [−10.04–2.58]	−4.25 [−9.89–1.39]
Divers or missing	−5.73 [−23.57–12.10]	−8.15 [−25.59–9.29]
Hospital owned by a private healthcare company	Reference	Reference
Community Hospital	4.35 [−1.12–9.82]	4.12 [−2.68–10.92]
University Hospital	2.35 [−2.69–7.39]	2.68 [−4.18–9.54]
**PSC-12 Change T0–T2**		
Intercept	**11.65 [8.42–14.88]**	**9.11 [5.13–13.09]**
**PSC-12 at T0**	**−0.44 [−0.5184 – −0.36]**	**−0.32 [−0.42 – −0.22]**
Group (IG)	0.59 [−0.94–2.12]	0.43 [−1.53–2.39]
Group (WCG)	Reference	Reference
Hierarchy level (top management)	**4.43 [0.98–7.88]**	**4.76 [1.13–8.39]**
Hierarchy level (middle management)	**2.38 [0.81–3.95]**	**2.45 [0.65–4.25]**
Hierarchy level (no management)	Reference	Reference
Male	Reference	Reference
Female	0.3 [−1.54–2.14]	0.11 [−1.89–2.11]
Divers or missing	−0.49 [−5.37–4.39]	–
Hospital owned by a private healthcare company	Reference	Reference
Community Hospital	−0.99 [−2.85–0.87]	−1.58 [−4.11–0.95]
University Hospital	−1.13 [−3.09–0.83]	−1.4 [−3.99–1.19]

Notes: mITT = modified intention to treat; CI = confidence interval; PP = per protocol; models.

A sensitivity analysis based on the complete mITT cases (i.e., without imputation) revealed partially divergent estimates and significance levels, suggesting that the imputation model may have impacted the results. The complete case results are presented in S2 Table. Complete mITT data without imputation were available for N = 253 participants for the primary outcome (IRR) in the sensitivity analysis. Regarding the secondary outcomes, N = 246 participants provided complete data for the WHO-5 and N = 251 participants provided complete data for the PSC-12.

In summary, the multilevel intervention showed no effect on the change score in the primary (IRR) or secondary (WHO-5; PSC-12) outcomes in either the mITT or PP set (see Table 3). In all ANCOVA models, baseline score at T0 was a significant predictor of the corresponding change score. Neither the study site, gender nor age had a statistically significant association with any of the three outcomes in either the mITT or PP ANCOVA models. Hierarchy level was statistically significantly different, with 2.4 points (middle management) or 4.4 points (top management) having a greater change in psychosocial safety score change (T2 minus T0), indicating an average increase at T2 in these groups compared to the participants with no responsibility (all else being held equal). Similarly, the sensitivity analysis showed no statistically significant effect on any of the three outcomes in the PP set.

## Discussion

The present study found no effect of a multilevel, individual and organizational intervention on staff irritation, mental well-being or the psychosocial safety climate between baseline and 11-month follow-up. Although the main ANCOVA models based on imputed data revealed no significant group differences, a sensitivity analysis using only complete mITT cases produced largely consistent results for the WHO-5 and PSC-12 outcomes. However, the IRR score estimate indicated an opposite effect compared to the imputed model, suggesting that the imputation process may have influenced this particular result.

The absence of intervention effects must also be considered within the context of the overall psychosocial environment at baseline, because it could have impacted the implementation process and the outcomes. The psychosocial safety risk context at baseline must also be considered. According to the PSC benchmark risk levels [46], participants in the present trial were classified as high risk (i.e., experiencing low levels of PSC) for future depressive symptoms and job strain at at T0. The average PSC-12 total score was M = 29.1 (SD = 9.7). While this value does not fall into the “very high risk” category (PSC ≤ 26), it does fall within the “high risk” range (PSC 27–36). This range is still associated with an increased likelihood of impaired mental health and workplace stress. These results suggest that participating hospital staff worked under psychosocial conditions that were clearly worse than those observed in reference populations. This supports the need for structural and preventive interventions and validates the decision to implement targeted training in this context.

Furthermore, potential contamination effects should be considered. It is possible that individuals who did not actively participate in the intervention were indirectly influenced, for example, by changes in leadership style or altered team dynamics. These spillover effects could have reduced between-group differences, especially regarding organizational-level outcomes.

The present findings for this multilevel intervention targeting mental health problems/stress prevention in the hospital setting are therefore partly inconsistent with previous studies reporting more positive or mixed effects: For example, in a subgroup analysis of their meta-analysis, one study [19] considered organizational interventions to be more effective and necessary to reduce the risk of physician burnout than individual interventions. However, other purely organizational interventions to change working conditions were also found to be ineffective among healthcare workers [11].

West et al. [51] randomized 74 out of 565 practicing physicians, which corresponds to a participation rate of 13%. In addition, most of the described intervention studies included a specific group of hospital workers (mainly doctors) in the study population [51,52]. In this study, all hospital staff were included in the study population. Therefore, the results are not easily comparable. In general, possible reasons for lower participation in workplace health promotion are, for example, programmes during leisure time, low social support, very strenuous work and high physical or emotional demands combined with little control over work [53]. These reasons seem to appear to be particularly virulent in the hospital workplace. Another cluster randomized control trial reported similar difficulties and barriers [22]. The associated process analysis showed that almost 70% of participants felt that the intervention had made no difference to their working conditions. In our study, similar to Montano [22] and West [51], we were only able to reach a minority, about 8% of the eligible health professionals. In terms of the overall recruitment process and the participation in the modules of this study, the most common challenges can be summarized as follows: A large proportion of potential participants reported an ongoing stressful situation, which subjectively made it almost impossible to participate in training. Perceived time pressure and staff shortages in daily clinical practice were often cited as the main factors. This also points to structural problems in the German clinical health care system that lead to a demotivation of hospital staff and a structural inability to participate in health-related interventions [54].

Comparisons of our results with existing evidence should also take into account differences in the intensity and duration of interventions and target groups. There is considerable variation in the dose and duration of interventions in different healthcare settings. For example, Weigl et al. [52] delivered continuous small group meetings for 10 months (11 x 1.5 hours). West et al. [51] evaluated 19 biweekly facilitated small group discussions over 9 months. The maximum duration of the seminars in our study (12 hours), which was a limitation imposed by the hospital administration, may reflect the scarce resources and difficult working conditions in hospitals. Therefore, future studies should apply a higher overall dose of interventions, especially with more continuous/ repeated training sessions that allow participants to engage in an individual stepwise learning process.

In addition, structural and rather frequent changes in staff and management at the hospital studied during the intervention phase may have influenced the present results. For example, the construction and relocation of a new hospital meant that the local management had other urgent priorities. In addition, two study sites underwent a complete restructuring due to the merger of a hospital group, which also resulted in a change of hospital management and the board of directors. Furthermore, one potential explanation for the limited intervention effects may be the relatively low level of active involvement by key stakeholders, particularly hospital management. Although the intervention addressed organizational-level factors, the engagement of leadership throughout the planning, communication and implementation phases appeared limited. Previous research has emphasized that manager involvement is a critical factor for the success of workplace interventions and that its absence is a known barrier to effectiveness [55–57]. Future implementation efforts should therefore systematically involve leadership in all stages of intervention design and delivery to increase organizational ownership and effectiveness.

Given the promising results of the pilot interventions (Phase I of the SEEGEN trial), the lack of significant effects in the current trial merits further consideration. The pilot interventions showed positive outcomes, such as increased self-efficacy, reduced irritation, and improved leadership behaviors. However, these effects may not have been replicated on a larger scale due to several factors. Implementing the interventions across multiple hospital sites likely introduced greater heterogeneity in terms of context and staff engagement. Additionally, the onset of the SARS-CoV-2 pandemic disrupted continuity of the interventions, necessitated a shift to online formats, and challenged organizational capacities – factors that may have diluted the impact of the interventions.

These findings suggest that a) specific feasibility studies and b) specifically targeted, phased and multi-level strategies prepared over a longer period of time are needed to motivate the majority of hospital workers to participate in such individual and organizational interventions.

The lower than planned participation rate in the modules may also be partly due to the significant and sustained impact of the SARS-CoV-2 pandemic on hospital workers [58], which started in the middle of our intervention phase. In addition, pandemic-related changes in the work environment, such as increased work intensification or task concentration due to redeployment or temporary transfers, were observed by the local study teams and may have made participation in the offered modules more difficult.

### Limitations

Several limitations of the study should be noted. First, the study was not conducted entirely as planned due to the global SARS-CoV-2 pandemic and the associated lockdown periods, which greatly increased work demands and changed the work environment. The study had to be adapted several times to the rapidly evolving pandemic situation. Both, the post- and follow-up surveys were conducted in the middle (T1 at the end of the Wave I-related lockdown of SARS-CoV-2 pandemic in Germany) and at the peak of the SARS-CoV-2 pandemic (T2 during the Wave II-related lockdown of SARS-CoV-2 pandemic). On top of all the other pre-existing stressors, this created an exceptional situation on the wards in terms of patient care and hospital management. Priorities were further shifted towards crisis management. The psychosocial burden for almost all hospital employers was at a peak at this time [58], which may also have contributed to the failure of the multilevel intervention to show improvements in the primary and secondary endpoints. As a minor point, the content of the workshops was not adapted to the burden of the SARS-CoV-2 pandemic after its outbreak in the middle of our intervention phase, in order to offer the same workshops to all participants. This may have resulted in participants finding the content less relevant at that time. Secondly, the study did not reach the planned sample size (415 out of 720 or 58%). This limited recruitment may affect the generalizability and statistical power of the findings and should be considered when interpreting the results. Thirdly, the low dose and limited acceptance of the intervention may have made it impossible for participants to engage in an ongoing dynamic process of individual and group development. The mediators and moderators of the effect mechanisms of action will be the subject of a forthcoming publication [59]. Although our primary and secondary outcome measures were ordinal in nature, we applied parametric models (e.g., ANCOVA), which is a common approach in similar trials. However, this approach may introduce limitations regarding the precision and assumptions of the statistical estimates, so the results should be interpreted accordingly.

A potential limitation of our analysis is the relatively high proportion of missing data (approximately 40%). However, multiple imputation is widely recommended for handling missing data in clinical and epidemiological studies, even with modest sample sizes, as long as the missing at random (MAR) assumption is plausible [47]. Furthermore, sensitivity analyses based on complete-case data produced consistent results, indicating that our findings are robust with respect to the imputation strategy.

Despite the absence of statistically significant effects, the study has several methodological strengths. It provides rare, empirical insights from a large-scale, multicenter cluster-randomized trial in the complex and under-researched hospital setting, a context in which workplace mental health interventions are rarely studied. Therefore, the study contributes to implementation and feasibility research in this field.

## Conclusions

This cRCT revealed that the SEEGEN intervention did not substantially reduce occupational stress or improve the well-being of hospital workers. Statistically significant improvements were not observed in any of the primary or secondary outcomes (IRR, WHO-5, or PSC-12), highlighting the limited effectiveness of the SEEGEN intervention under real-world conditions. Implementation was severely limited by external factors, including limited resources and the SARS-CoV-2 pandemic. Therefore, under the tested conditions and in its current form, the intervention cannot be recommended for broader practice.

### Implications and future research directions

Although there were no statistically significant effects, this study provides valuable insights for the real-world implementation of multilevel workplace interventions. The findings underscore the importance of organizational readiness, adequate resource allocation, and dedicated time for staff to engage with change processes. Interventions must be flexible enough to adapt to external pressures, such as staffing shortages or public health crises. In practice, future efforts should focus on strengthening the conditions that enable meaningful participation and long-term integration into routine practice, as well as on intervention content.

Future studies should address the identified limitations to enable implementation under improved conditions. This includes ensuring sufficient organizational support, a higher intervention dosage, and protected time for participation. Furthermore, future evaluations should investigate whether the lack of effect reflects true ineffectiveness or is primarily due to implementation barriers under crisis conditions.

## Supporting information

S1 Table CONSORT 2010 checklist of information to include when reporting a cluster randomized trial. (DOCX)

S2 Table Sensitivity analysis of primary and secondary outcomes (ANCOVA model based on complete mITT set). (DOCX)

S1 Document Clinical trial protocol. (DOCX)

S2 Document Ethics application (in original language). (DOCX)

S3 Document English translation of the ethics application. (DOCX)
