# Supplementary material for: Effectiveness of a multilevel intervention to improve mental health of hospital workers: The SEEGEN multicenter cluster randomized controlled trial
Source: PLoS One. 2025 Aug 21;20(8):e0330490. doi: 10.1371/journal.pone.0330490 (PMC12370060; doi:10.1371/journal.pone.0330490)
Supplement: S1 Table — (DOCX) [file pone.0330490.s001.docx]

**S1 Table.** **CONSORT 2010 checklist of information to include when reporting a cluster randomized trial**

| Section/Topic | Item N° | Standard checklist item | Extension for cluster designs | Page N°* |
| --- | --- | --- | --- | --- |
| Title and abstract | | | |  |
|  | 1a | Identification as a randomized trial in the title | Identification as a cluster randomized trial in the title | 1, 2 |
|  | 1b | Structured summary of trial design, methods, results, and conclusions (for specific guidance see CONSORT for abstracts)^[[1]](#endnote-1),^^[[2]](#endnote-2)^ | See Table 2 | 2-3 |
| Introduction | | | |  |
| Background and objectives | 2a | Scientific background and explanation of rationale | Rationale for using a cluster design | 4-7 |
|  | 2b | Specific objectives or hypotheses | Whether objectives pertain to the cluster level, the individual participant level or both | 7 |
| Methods | | | |  |
| Trial design | 3a | Description of trial design (such as parallel, factorial) including allocation ratio | Definition of cluster and description of how the design features apply to the clusters | 7/8 |
|  | 3b | Important changes to methods after trial commencement (such as eligibility criteria), with reasons |  | 9/10 |
| Participants | 4a | Eligibility criteria for participants | Eligibility criteria for clusters | 8 |
|  | 4b | Settings and locations of data collection |  | 8/9 |
| Interventions | 5 | The interventions for each group with sufficient details to allow replication, including how and when they were actually administered | Whether interventions pertain to the cluster level, the individual participant level or both | 10ff |
| Outcomes | 6a | Completely defined pre-specified primary and secondary outcome measures, including how and when they were assessed | Whether outcome measures pertain to the cluster level, the individual participant level or both | 16/17 |
|  | 6b | Any changes to trial outcomes after the trial commenced, with reasons |  | No changes to trial outcomes were made. |
| Sample size | 7a | How sample size was determined | Method of calculation, number of clusters(s) (and whether equal or unequal cluster sizes are assumed), cluster size, a coefficient of intracluster correlation (ICC or *k*), and an indication of its uncertainty | 17 |
|  | 7b | When applicable, explanation of any interim analyses and stopping guidelines |  | No interim or early stopping rules were planned or implemented for this study. The trial ran to its planned conclusion. Therefore, this item is not applicable. |
| Randomization: | | | |  |
| Sequence generation | 8a | Method used to generate the random allocation sequence |  | 17/18 |
|  | 8b | Type of randomization; details of any restriction (such as blocking and block size) | Details of stratification or matching if used | 17/18 |
| Allocation concealment mechanism | 9 | Mechanism used to implement the random allocation sequence (such as sequentially numbered containers), describing any steps taken to conceal the sequence until interventions were assigned | Specification that allocation was based on clusters rather than individuals and whether allocation concealment (if any) was at cluster level, individual participant level or both | 17/18 |
| Implementation | 10 | Who generated the random allocation sequence, who enrolled participants, and who assigned participants to interventions | Replace by 10a, 10b and 10c | 8; 17/18 |
|  | 10a |  | Who generated the random allocation sequence, who enrolled clusters, and who assigned clusters to interventions |  |
|  | 10b |  | Mechanism by which individual participants were included in clusters for the purposes of the trial (such as complete enumeration, random sampling) |  |
|  | 10c |  | From whom consent was sought (representatives of the cluster, or individual cluster members, or both), and whether consent was sought before or after randomization |  |
|  |  |  |  |  |
| Blinding | 11a | If done, who was blinded after assignment to interventions (for example, participants, care providers, those assessing outcomes) and how |  | Blinding was not feasible due to the nature of the intervention, which was organizational, multilevel, and visibly implemented within teams. Participants and facilitators necessarily knew which group they were assigned to. |
|  | 11b | If relevant, description of the similarity of interventions |  | Although a waitlist control group was used, its participants did not receive any form of treatment during the trial period. Maintaining similarity to support blinding was not relevant. |
| Statistical methods | 12a | Statistical methods used to compare groups for primary and secondary outcomes | How clustering was taken into account | 18ff |
|  | 12b | Methods for additional analyses, such as subgroup analyses and adjusted analyses |  | No additional or subgroup analyses were conducted; everything was described a priori in the published study protocol. |
| Results | | | |  |
| Participant flow (a diagram is strongly recommended) | 13a | For each group, the numbers of participants who were randomly assigned, received intended treatment, and were analysed for the primary outcome | For each group, the numbers of clusters that were randomly assigned, received intended treatment, and were analysed for the primary outcome | 22ff; Fig. 1 |
|  | 13b | For each group, losses and exclusions after randomization, together with reasons | For each group, losses and exclusions for both clusters and individual cluster members | 22; Fig. 1 |
| Recruitment | 14a | Dates defining the periods of recruitment and follow-up |  | 7/8 |
|  | 14b | Why the trial ended or was stopped |  | The trial was conducted and completed as planned. No early termination or stopping rules were applied. |
| Baseline data | 15 | A table showing baseline demographic and clinical characteristics for each group | Baseline characteristics for the individual and cluster levels as applicable for each group | Table 1 site 23 |
| Numbers analysed | 16 | For each group, number of participants (denominator) included in each analysis and whether the analysis was by originally assigned groups | For each group, number of clusters included in each analysis | 22ff; Fig. 1 |
| Outcomes and estimation | 17a | For each primary and secondary outcome, results for each group, and the estimated effect size and its precision (such as 95% confidence interval) | Results at the individual or cluster level as applicable and a coefficient of intracluster correlation (ICC or k) for each primary outcome | 24ff |
|  | 17b | For binary outcomes, presentation of both absolute and relative effect sizes is recommended |  | The study exclusively reported on continuous outcomes (IRR, WHO-5 and PSC-12) rather than binary endpoints. Therefore, this recommendation does not apply. |
| Ancillary analyses | 18 | Results of any other analyses performed, including subgroup analyses and adjusted analyses, distinguishing pre-specified from exploratory |  | As outlined in the study protocol, no additional analyses were conducted beyond the pre-specified primary and secondary analyses. |
| Harms | 19 | All important harms or unintended effects in each group (for specific guidance see CONSORT for harms^[[3]](#endnote-3)^) |  | No harmful or unintended effects were expected or observed. The intervention involved non-invasive organizational and training measures that posed no physical or psychological risk to participants. Therefore, this recommendation does not apply. |
| Discussion | | | |  |
| Limitations | 20 | Trial limitations, addressing sources of potential bias, imprecision, and, if relevant, multiplicity of analyses |  | 30/31 |
| Generalisability | 21 | Generalisability (external validity, applicability) of the trial findings | Generalisability to clusters and/or individual participants (as relevant) | The intervention did not yield significant results. Therefore, it is not possible to draw conclusions about the generalizability of the results to other clusters or individuals. |
| Interpretation | 22 | Interpretation consistent with results, balancing benefits and harms, and considering other relevant evidence |  | 27ff |
| Other information | | |  |  |
| Registration | 23 | Registration number and name of trial registry |  | 9 |
| Protocol | 24 | Where the full trial protocol can be accessed, if available |  | 9 |
| Funding | 25 | Sources of funding and other support (such as supply of drugs), role of funders |  | Not included in the manuscript, can be found in the submission system. |

1. [↑](#endnote-ref-1)
2. [↑](#endnote-ref-2)
3. [↑](#endnote-ref-3)
