# Supplementary material for: Effectiveness of a multilevel intervention to improve mental health of hospital workers: The SEEGEN multicenter cluster randomized controlled trial
Source: PLoS One. 2025 Aug 21;20(8):e0330490. doi: 10.1371/journal.pone.0330490 (PMC12370060; doi:10.1371/journal.pone.0330490)
Supplement: S2 Table — (DOCX) [file pone.0330490.s002.docx]

**S2 Table. Sensitivity analysis of primary and secondary outcomes (ANCOVA model based on complete mITT set)**

|  | **Complete mITT set (N=253)** |
| --- | --- |
| **IRR Change T0–T2** | Estimate (95%CI) |
| Intercept | -7.07 [-11.34, -2.80] |
| Group (IG) | 1.09 [-1.09, 3.27] |
| Group (WCG) | Reference |
| Hierarchy level (top management) | -1.17 [-5.29, 2.95] |
| Hierarchy level (middle management) | 0.08 [-1.94, 2.10] |
| Hierarchy level (no management) | Reference |
| Male | Reference |
| Female | -0.64 [-2.91, 1.63] |
| Hospital owned by a private healthcare company | Reference |
| Community Hospital | 0.18 [-2.56, 2.92] |
| University Hospital | -0.77 [-3.67, 2.13] |
| **WHO-5 Change T0–T2** | (N=246) |
| Intercept | 11.80 [1.53, 22.07] |
| Group (IG) | 2.69 [-2.54, 7.92] |
| Group (WCG) | Reference |
| Hierarchy level (top management) | -2.64 [-11.87, 6.59] |
| Hierarchy level (middle management) | -0.74 [-5.31, 3.83] |
| Hierarchy level (no management) | Reference |
| Male | Reference |
| Female | -4.38 [-9.53, 0.77] |
| Hospital owned by a private healthcare company | Reference |
| Community Hospital | 5.81 [-0.70, 12.32] |
| University Hospital | 3.52 [-3.26, 10.30] |
| **PSC-12 Change T0–T2** | **(N=251)** |
| Intercept | 9.11 [5.13, 13.09] |
| Group (IG) | 0.43 [-1.53, 2.39] |
| Group (WCG) | Reference |
| Hierarchy level (top management) | 4.76 [1.13, 8.39] |
| Hierarchy level (middle management) | 2.45 [0.65, 4.25] |
| Hierarchy level (no management) | Reference |
| Male | Reference |
| Female | 0.11 [-1.89, 2.11] |
| Hospital owned by a private healthcare company | Reference |
| Community Hospital | -1.58 [-4.11, 0.95] |
| University Hospital | -1.40 [-3.99, 1.19] |
