# Supplementary material for: Effectiveness of a multilevel intervention to improve mental health of hospital workers: The SEEGEN multicenter cluster randomized controlled trial
Source: PLoS One. 2025 Aug 21;20(8):e0330490. doi: 10.1371/journal.pone.0330490 (PMC12370060; doi:10.1371/journal.pone.0330490)
Supplement: S1 Document — (DOCX) [file pone.0330490.s003.docx]

30.10.2020 / Version number 5.0

**PROTOCOL**

**A cluster-randomized trial evaluating a complex intervention to improve mental health and well-being of employees working in hospital – the SEEGEN trial**

**[DRKS00017249]**

# Table of contents

[Table of contents 2](#_Toc54689571)

[1. Trial Team 4](#_Toc54689572)

[2. Coinvestigators / Principal Investigators 5](#_Toc54689573)

[3. Protocol Summary 7](#_Toc54689574)

[Figure 1: Flow Diagram 11](#_Toc54689575)

[Table 1: Trial schedule 12](#_Toc54689576)

[Abbreviations and Definitions 14](#_Toc54689577)

[4. Introduction and Study Rationale 15](#_Toc54689578)

[5. Trial Objectives and Purpose 16](#_Toc54689579)

[5.1. Primary objective 16](#_Toc54689580)

[5.2. Secondary objectives 16](#_Toc54689581)

[5.3. Process evaluation 17](#_Toc54689582)

[6. Outcomes 18](#_Toc54689583)

[6.1. Primary Outcome 18](#_Toc54689584)

[6.2. Secondary Outcomes 18](#_Toc54689585)

[6.2.1. Main secondary outcomes 18](#_Toc54689586)

[6.2.2. Further secondary outcomes 18](#_Toc54689587)

[6.3. Process measures 22](#_Toc54689588)

[7. Modules of the complex intervention 22](#_Toc54689589)

[7.1. Top Management Training 22](#_Toc54689590)

[7.2. Promoting stress preventive relational leadership competence 23](#_Toc54689591)

[7.3. Dilemma Competency – Coping by taking responsibility 23](#_Toc54689592)

[7.4. Reconciling Work and Family Life 24](#_Toc54689593)

[7.5. Stay Healthy at Work 24](#_Toc54689594)

[7.6. Roundtable discussions and booster sessions 24](#_Toc54689595)

[8. Design of the Trial 25](#_Toc54689596)

[8.1. Trial Design 25](#_Toc54689597)

[8.2. Randomisation and Blinding 25](#_Toc54689598)

[8.3. Additional Methods for Reducing Bias 25](#_Toc54689599)

[8.4. Duration of Trial Period(s) 25](#_Toc54689600)

[9. Study population and withdrawal of subjects 26](#_Toc54689601)

[9.1. Number of Subjects 26](#_Toc54689602)

[9.2. Subject Inclusion Criteria 26](#_Toc54689603)

[9.3. Subject Exclusion Criteria 26](#_Toc54689604)

[9.4. Subject Withdrawal Criteria 26](#_Toc54689605)

[10. Study process 26](#_Toc54689606)

[11. Safety 27](#_Toc54689607)

[12. Data Collection, Data Management and Archiving 27](#_Toc54689608)

[12.1. Data collection 27](#_Toc54689609)

[12.2. Data Management 27](#_Toc54689610)

[12.3. Archiving 28](#_Toc54689611)

[13. Statistics 28](#_Toc54689612)

[13.1. Sample Size Calculation 28](#_Toc54689613)

[13.2. Statistical Methods 29](#_Toc54689614)

[13.3. Handling of Missing and Spurious Data and Drop outs 30](#_Toc54689615)

[14. Ethical and Legal Aspects 30](#_Toc54689616)

[14.1. Subject Information and Informed Consent 30](#_Toc54689617)

[14.2. Confidentiality 30](#_Toc54689618)

[14.3. Protocol Amendments 31](#_Toc54689619)

[14.4. Approval of the Clinical Trial Protocol and Amendments 31](#_Toc54689620)

[14.5. Ongoing Information of Independent Ethics Committee (IEC) 31](#_Toc54689621)

[14.6. Liability and Insurance 31](#_Toc54689622)

[15. Declarations 32](#_Toc54689623)

[16. References 33](#_Toc54689624)

# Trial Team

| **Sponsor** | **Study coordinator** |
| --- | --- |
| Federal Ministry of Education and Research, German Aerospace Center (DLR) Project Management Health | Prof. Dr. Harald Gündel |
| Heinrich-Konen-Straße 1, 53227 Bonn | Clinic of Psychosomatic Medicine and Psychotherapy, University Hospital Ulm  Albert-Einstein-Allee 23, 89081 Ulm |
| +49 (0)228 99 57 0 / +49 (0)228 99 57 83601 /  [information@bmbf.bund.de](mailto:information@bmbf.bund.de) | +49 (0)731 500-61801 / +49 (0)731 500-61802 /  harald.guendel@uniklinik-ulm.de |
|  |  |
| **Coordination of process evaluation** | **Head of research DKI / executive committee DKI** |
| Prof. Dr. phil. Bernd Puschner | Dr. PH Karl Blum |
| Department of Psychiatry and Psychotherapy II  Ulm University and Bezirkskrankenhaus Günzburg  Ludwig-Heilmeyer-Str. 2, 89312 Günzburg | Deutsches Krankenhausinstitut (DKI) e.V.  Hansaallee 201, Haus 1, 40549 Düsseldorf |
| +49 (0)8221 / 96-2866 / +49 (0)8221 / 96-28160/  bernd.puschner@bkh-guenzburg.de | +49 (0)211 47051-17 / 0211 47051-19 /  karl.blum@dki.de |
|  |  |
| **Biometrician** | **Data management** |
| Dr. Regina Krisam | Ronald Limprecht |
| Institute of Medical Biometry and Informatics  Department of Medical Biometry  University Hospital Heidelberg  Im Neuenheimer Feld 130.3 (Turm West), 69120 Heidelberg | Institute of Medical Biometry and Informatics  Department of Medical Biometry  University Hospital Heidelberg  Im Neuenheimer Feld 130.3 (Turm West), 69120 Heidelberg |
| +49 (0) 6221-56-35036/ +49 (0)6221-56-4195 /  [brinster@imbi.uni-heidelberg.de](mailto:brinster@imbi.uni-heidelberg.de) | +49 (0)6221-56-5500 / +49 (0)6221-56-4195 /  limprecht@imbi.uni-heidelberg.de |
|  |  |
| **Representative Biometrician** | **Methodological consulting** |
| Dr. sc. hum. Anja Sander | Dr. sc. hum. Marc N. Jarczok |
| Institute of Medical Biometry and Informatics  Department of Medical Biometry  University Hospital Heidelberg  Im Neuenheimer Feld 130.3 (Turm West), 69120 Heidelberg | Clinic of Psychosomatic Medicine and Psychotherapy,  University Hospital Ulm  Albert-Einstein-Allee 23, 89081 Ulm |
| +49 (0)6221-56-5859 / +49 (0)6221-56-4195 /  [sander@imbi.uni-heidelberg.de](mailto:sander@imbi.uni-heidelberg.de) | +49 (0)731 500 – 61810 / +49 (0)731 500 - 61802  marc.jarczok@uniklinik-ulm.de |
|  |  |
| **Project/trial manager** |  |
| Dr. biol. hum. Nadine Mulfinger |  |
| Clinic of Psychosomatic Medicine and Psychotherapy,  University Hospital Ulm  Albert-Einstein-Allee 23, 89081 Ulm |  |
| +49 (0)731 500- 61872 / +49 (0)731 500- 61802, nadine.mulfinger@uni-ulm.de |  |

# Coinvestigators / Principal Investigators

| **Düsseldorf** | **Duisburg-Essen** |
| --- | --- |
| Prof. Dr. med. Peter Angerer | Prof. Dr. phil. Andreas Müller |
| [Institute of Occupational and Social Medicine](https://www.uniklinik-duesseldorf.de/en/unternehmen/institute/institute-of-occupational-and-social-medicine/), University  Hospital Düsseldorf  Universitätsstraße 1, 40225 Düsseldorf | Faculty of Educational Sciences, Work and Organizational  Psychology  University Duisburg-Essen  Universitätsstr. 2  45141 Essen |
| +49 (0)211 - 81 14721 / +49 (0)211 - 81 15334  [peter.angerer@uni-duesseldorf.de](mailto:peter.angerer@uni-duesseldorf.de) | +49 (0)201-183-3613 / +49 (0)201-183-4350  andreas_mueller@uni-due.de |
|  |  |
| **Heidelberg** | **Tübingen** |
| Prof. Dr. rer. soc. Jochen Schweitzer-Rothers | Prof. Dr. med. Stephan Zipfel |
| Institute for Medical Psychology, University Hospital Heidelberg  Bergheimer Straße 20, 69115 Heidelberg | Clinic of Psychosomatic Medicine and Psychotherapy,  University Hospital Tübingen,  Osianderstraße 5, 72076 Tübingen |
| +49 (0)6221-56-8152 / +49 (0)6221-565303  [jochen.schweitzer-rothers@med.uni-heidelberg.de](mailto:jochen.schweitzer-rothers@med.uni-heidelberg.de) | +49 (0)7071 29-86714/ +49 (0)7071 29-4541  stephan.zipfel@med.uni-tuebingen.de |
|  |  |
| **Tübingen** | **Tübingen** |
| Dr. med. Florian Junne | Prof. Dr. med. Monika A. Rieger |
| Clinic of Psychosomatic Medicine and Psychotherapy,  University Hospital Tübingen,  Osianderstraße 5, 72076 Tübingen | Institute for Occupational Medicine, Social Medicine and Health  Services Research, University Hospital Tübingen  Wilhelmstraße 27, 72074 Tübingen |
| +49 (0)7071 29-86884 / +49 (0)7071 29-4541  florian.junne@med.uni-tuebingen.de | +49 (0)7071 29-82083 / +49 (0)7071 29-4362  monika.rieger@med.uni-tuebingen.de |
|  |  |
| **Ulm** | **Ulm** |
| Dr. med. Eva Rothermund | Prof. Dr. phil. Ute Ziegenhain |
| Leadership Personality Center Ulm (LPCU) Ulm University  Kornhausgasse 8, 89073 Ulm | Clinic of Child- and Adolescents Psychiatry / Psychotherapy,  University Hospital Ulm  Steinhövelstraße 3, 89075 Ulm |
| +49 (0)731 37991-501 / +49 (0)731 37991-510 /  [eva.rothermund@uni-ulm.de](mailto:eva.rothermund@uni-ulm.de) | +49 (0)731 500 – 61666 / +49 (0)731 500 - 61682  ute.ziegenhain@uniklinik-ulm.de |
|  |  |
| **Heidelberg** | **Heidelberg** |
| Dr. med. Imad Maatouk | Prof. Dr. med. Christoph Nikendei |
| Clinic of General Internal Medicine and Psychosomatic,  University Hospital Heidelberg  Im Neuenheimer Feld 410, 69120 Heidelberg | Clinic of General Internal Medicine and Psychosomatic,  University Hospital Heidelberg  Thibautstrasse 4  69115 Heidelberg |
| +49 (0)6221 56-37585 / +49 (0)6221 56 5749  Imad.Maatouk@med.uni-heidelberg.de | +49 (0)6221-5638663/ +49 (0)6221-565330  christoph.nikendei@med.uni-heidelberg.de |
|  |  |
| **Düsseldorf** | **Düsseldorf** |
| Prof. Dr. rer. pol. Stefan Süß | Dr. rer. oec. Sascha Ruhle |
| Institute of Business Administration, esp. Organization and Personnel,  Heinrich-Heine-University Düsseldorf  Universitätsstraße 1, 40225 Düsseldorf | Institute of Business Administration, esp. Organization and Personnel,  Heinrich-Heine-University Düsseldorf  Universitätsstraße 1, 40225 Düsseldorf |
| +49 (0)211 81 13 995/+49 (0)211 81 15 164  [stefan.suess@hhu.de](mailto:stefan.suess@hhu.de) | +49 (0)211 – 8110249 / +49 (0)211-8115164  sascha.ruhle@hhu.de |
|  |  |

# Protocol Summary

1. **Title**

A cluster-randomized trial evaluating a complex intervention to improve mental health and well-being of employees working in hospital – the SEEGEN trial

1. **Study Sites**

3 study locations (Aalen/Ellwangen, Heidelberg, Duisburg) with varying number of participating clusters

1. **Phase**

Confirmatory phase III trial

1. **Rationale**

Hospital employees face many challenges and stress in their daily work life, which can lead to impaired health and well-being. However, evidence-based programs to improve mental health and well-being of hospital employees are rare. SEEGEN develops and tests the effectiveness of a complex intervention to improve work-related stressors in hospital employees.

1. **Objectives**

To evaluate the effect of a complex intervention on mental health and well-being of employees working in hospital.

1. **Design**

Open cluster-randomized trial with waitlist control group

1. **Study Population**

Hospital employees at different levels of hierarchy

1. **Sample Size**

Total sample size of 720 study participants (intervention and waitlist control group)

1. **Intervention**

Hospital employees can choose one of the following modules that aim to improve managing work-related challenges: For the top management a management training to raise awareness regarding the design of health-promoting work will be offered, for all other occupational groups in leadership position a management training to strengthen the relational and stress preventive leadership competence will be offered. For all those who are confronted with a dilemma situation regardless of their professional position, a dilemma competency training will be provided. Particular attention will be given to the different life phases of the employees: While one module deals with reconciling work and family life, another module covers the topic staying healthy in hospital. The effectiveness of the modules will be assessed by various questionnaires completed by the study participants (intervention and waitlist control group). In addition, employees who do not participate in the modules but wish to participate in the SEEGEN study by answering shorter questionnaires (cluster participants) are included in the trial to measure dispersal effects of the SEEGEN study.

The outbreak of the COVID-19 pandemic led to an interruption of the complex intervention and thus made changes in the study design as well as an adjustment of the questionnaires necessary. Before restarting the complex intervention, a questionnaire (including the instruments described) will be sent to the study participants (T1*). Additional questions on the COVID-19 pandemic will be included to evaluate the mental health of hospital workers also in relation to the pandemic. At the end of the complex intervention, this questionnaire is sent out again to the study participants (T2*).

1. **Primary Endpoint(s)**

The change in the total score of the Irritation scale (IRR) according to Mohr et al. (2005) from baseline (T0) to T2* (eleven months after T0) between the intervention and waitlist control group.

1. **Secondary Endpoints**

**Main secondary outcomes:**

- Change in World Health Organization Well-Being Index (WHO-5 Well Being Index, Topp et al., 2015) from T0 to T2* between the intervention and waitlist control group.
- Change in Psychosocial safety climate (PSC-12, G. B. Hall et al., 2010) from T0 to T2* between the intervention and waitlist control group.

**Further secondary outcomes:**

- Change in the total score of the IRR from T0 to 6 months after baseline (T1*) as well as the changes from T0 to T2* and T0 to T1* for both subscales between the intervention and waitlist control group.

The change in scores from T0 to T2*, as well as from T0 to T1*, between the intervention and waitlist control group of the following:

- World Health Organization Well-Being Index (WHO-5 Well-Being Index, Topp et al., 2015)
- Psychosocial safety climate (PSC-12; 12 items, G. B. Hall et al., 2010)
- Effort-Reward Imbalance Scale – Short version (ERI, 10 items, Siegrist et al., 2009)
- Work Analysis Instrument for Hospitals – Self-Report Version (2 subscales, 6 items, Glaser, 1997)
- Questionnaire on integrative leadership (FIF, 24 items) (Rowold & Poethke, 2017)
- Short version of the Occupational Self-Efficacy Scale (SOSES; 6 items, Rigotti et al., 2008)
- Top Management Evaluation Sheet (only for top management*)*
- Organizational indicators on individual/participant level (Job satisfaction, 8 items, Fischer & Lück, 2001; Employer attractiveness, 5 items, Bruhn et al., 2013, Presenteeism, 1 item, Preisendörfer, 2010; Intention to leave, 1 item, Simon et al., 2005; Recommendation, 1 item, modified version of Reichheld, 2003; Absenteeism; 1 item, modified version of Caverley et al., 2007; Work overtime; Economic situation of clinic/unit; 1 item, adapted version of A. Hall & Rohrbach-Schmidt, 2013; Job security; 1 item, Lee et al., 2004; Cooperation between occupational groups, 2 items, TAA-KH-S subscale social climate, Glaser, 1997)
- Organizational indicators on hospital/clinic level (Turnover rate, Number of applicants, Number of persons hired, Number of persons retired, Number of dismissals, Number of expiring fixed-term contracts, Absence rate, Work overtime rate, Sickness absence rate, Age distribution) requested from the participating hospitals’ accounting divisions

The change in scores from T1* to T2*, between the intervention and waitlist control group of the following:

- COVID-19 exposure and (mental) effects (concerns about COVID-19, trust in employer etc.; 12 items, five additional open questions)

The change in scores from T0 to T2*, as well as from T0 to T1*, between cluster participants (no module participation) of the following:

- IRR (Mohr et al., 2005)
- WHO-5 Well-Being Index (Topp et al., 2015)
- PSC-12 (G. B. Hall et al., 2010)
- Global Transformational Leadership (GTL; Carless et al., 2000)

The change in scores from T1* to T2*, between between cluster participants of the following:

- COVID-19 exposure and (mental) effects

In addition, the following outcomes will be collected at T2. The inclusion of these items enables to identify predictors for Work-Life-Balance:

- Personal data that provide information about the number of own children, childcare facilities and the partner's work activity (9 items),
- Trierer scale to measure Work-Life Balance (TKS-WLB, 5 items, Syrek et al., 2011) and
- The subscales “emotional demands” and “hiding emotions” of the Copenhagen Psychosocial Questionnaire (COPSOQ, 4 items Nübling, 2005).

1. **Statistical Methods**

The primary analysis will be performed according to the intention-to-treat principle comparing the change in the total score of IRR from baseline (T0) to 11 months after baseline (T2*) between the intervention group and the waitlist control group. To account for the clustering structure, an ANCOVA approach is used for analysis, including the treatment group, baseline value of the total score of the IRR at T0, hierarchy level, gender as well as the site as covariates and the respective cluster as a random effect. In case of significance, this approach is also used to hierarchically test for the secondary outcome, the change in the WHO-5 from T0 to T2*, in a confirmatory manner. Again, in case of significance the change in the PSC-12 from T0 to T2* will also be tested. All primary and secondary outcomes will also be analysed descriptively.

1. **Study Durations and Dates**

Total study duration [24 months]

Process evaluation: [28 months]

Duration of longest intervention: [10 months]

Duration of whole intervention and observation phase: [12 months]

Beginning of the preparation phase: [11/2018]

Start of recruitment: [10/2019]

End of recruitment: [03/2020]

End of observation phase: [03/2021]

Data base lock [06/2021]

End of waitlist control intervention: [04/2021]

Statistical analyses completed: [08/2021]

Study report completed: [09/2021]

# Figure 1: Flow Diagram


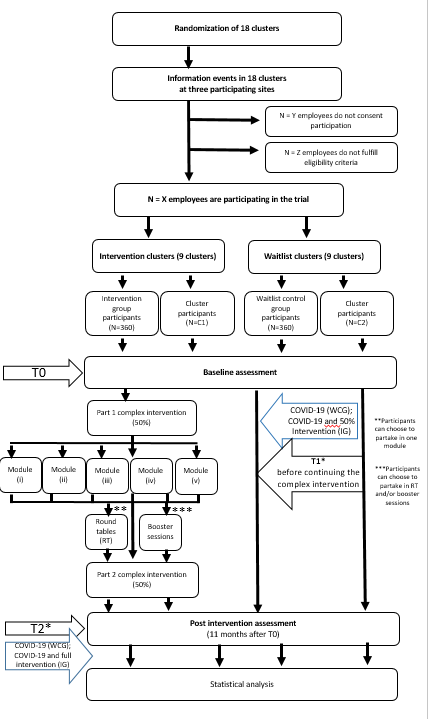


# Table 1: Trial schedule

|  | T0  (for intervention and wait list control group participants) | T0  (for cluster partici-pants) | T1*  (for intervention and wait list control group participants; 6 months after T0, ± 1 month) | T1*  (for cluster participants; 6 months after T0, ± 1 month) | T2*  (for intervention and wait list control group participants; (11 months after T0, ± 1 month) | T2*  (for cluster participants;11 months after T0; ± 1 month) |
| --- | --- | --- | --- | --- | --- | --- |
| Sociodemographic aspects | x | x |  |  |  |  |
| Inclusion and exclusion criteria | x | x |  |  |  |  |
|  |  |  |  |  |  |  |
| IRR | x | x | x | x | x | x |
| WHO-5 Well-Being Index | x | x | x | x | x | x |
| PSC-12 | x | x | x | x | x | x |
| ERI | x |  | x |  | x |  |
| TAA-KH S 8 | x |  | x |  | x |  |
| FIF, Modul A – transformational leadership | x |  | x |  | x |  |
| GTL |  | x |  | x |  | x |
| SOSES | x |  | x |  | x |  |
| Top Management Evaluation sheet | x |  | x |  | x |  |
| **Organizational indicators** |  |  |  |  |  |  |
| Job satisfaction | x |  | x |  | x |  |
| Employer attractiveness | x |  | x |  | x |  |
| Presenteeism | x |  | x |  | x |  |
| Intention to leave | x |  | x |  | x |  |
| Absenteeism | x |  | x |  | x |  |
| Recommendation | x |  | x |  | x |  |
| Work overtime | x |  | x |  | x |  |
| Economic situation of clinic/unit | x |  | x |  | x |  |
| Job security | x |  | x |  | x |  |
| Cooperation between occupational groups | x |  | x |  | x |  |
| Turnover | x |  | x |  | x |  |
| Absence rate | x |  | x |  | x |  |
| Work overtime rate | x |  | x |  | x |  |
| Sickness absence rate | x |  | x |  | x |  |
| Age distribution | x |  | x |  | x |  |
| **Process evaluation** |  |  |  |  |  |  |
| SEEGEN intervention fidelity scale |  |  | x |  |  |  |
| 17 items on exposure and (mental) effects caused by COVID-19 |  |  | x | x | x | x |
| 9 items on personal data (number of own children, childcare facilities and the partner's work activity) |  |  |  |  | x |  |
| Trierer Scale to measure Work-Life Balance (TKS-WLB) |  |  |  |  | x |  |
| COPSOQ |  |  |  |  | x |  |

# Abbreviations and Definitions

CRF Case Report Form

COPSOQ Copenhagen Psychosocial Questionnaire

DBL Database Lock

ERI Effort-Reward Imbalance

FIF Fragebogen zur integrativen Führung [Questionnaire for integrative leadership]

GCP Good Clinical Practice

GTL Global Transformational Leadership

HC Health Circle

ICH International Conference on Harmonisation of Technical Requirements for Registration of Pharmaceuticals for Human Use

IEC Independent Ethics Committee

IG Intervention Group

IMBI Institute of Medical Biometry and Informatics

IRR Irritation Scale

LKP Leiter der Klinischen Prüfung

PSC Psychosocial Safety Climate

SOC Selection, Optimization and Compensation

SOSES Short version of the Occupational Self-Efficacy Scale

TAA-KH-S Tätigkeits- und Arbeitsanalyseverfahren für das Krankenhaus

TKS-WLB Trierer Scale to measure Work-Life Balance

WCG Waitlist Control Group

# Introduction and Study Rationale

Health as well as the development and course of diseases are determined by very different factors. Many of those factors are particularly influenced by the professional environment. On the one hand, in a positive sense, e.g., by way of experiencing recognition, being creative and productive or establishing social contacts, which can help to cope with stressful situations. On the other hand, in a negative sense, e.g., in cases where the modern, compact world of work is very demanding and, thus, creates feelings of excess strain or isolation. Due to cost-cutting measures, working areas in hospitals and clinics have been subject to enormous changes over the last few years, especially with regard to the working conditions.

Hospital employees are a particularly vulnerable group because of their high occupational stress levels (Dollard et al., 2007). In a systematic review (Dollard et al., 2007), the individual and organisational impact of work-related stress in the Australian and international health and social services (HCS) sectors were examined. The results demonstrate high levels of occupational stress in the HCS sector. Work stress had a negative impact both on the individual (e.g. mental health) and on the organisation (e.g. absenteeism, job dissatisfaction).

Although health care workers show increased work-related stress and suicide rates (Agerbo et al., 2007), most of the hospitals in Germany lack of a systematic occupational health management system which links behavioural and organizational prevention. The high complexity of the hospital is often cited as a reason for this lack. As a reaction, often only occupational health promotion takes place in "campaignable" sub-areas or individual occupational groups (van Wyk & Pillay-Van Wyk, 2010). The evidence of individual intervention types has only been partially proven. In particular, the evaluation of combined behavioral and organizational interventions in the health care system is still outstanding (Ruotsalainen et al., 2015). In other sectors, some studies indicate an increased effectiveness of the combination of these two intervention types (Tetrick & Winslow, 2015). Existing reviews emphasize the need for methodologically high-quality studies in the field of health promotion in hospitals (Ruotsalainen et al., 2015; van Wyk & Pillay-Van Wyk, 2010).

The construct psychosocial safety climate (PSC) refers to policies, practices, and procedures that aim to protect workers psychological health and safety. According to Dollard and Bakker (2010), PSC is a facet-specific element of organizational climate, a ‘climate for psychosocial health and safety’, which is expected to precede working conditions.

A related construct represents the safety climate construct (Zohar, 2010).This construct focuses on policies and practices regarding safety and protection from physical hazards in the workplace, whereas psychological health and safety at work receive far less attention. The extension of the construct to psychological health seems important from a health and social policy perspective, as it can improve strategies and procedures to protect the mental health of employees (Dollard, 2012).

One of the major challenges in work stress research is to identify the origin of work stress so that interventions can be used as efficiently and effectively as possible. Existing research is characterized by work stress theories that focus on the conditions of job tasks as the origin of work stress. However, since companies are complex multi-layered systems (Mathieu & Taylor, 2007), the origin of work stress could lie in the dynamic interaction of different levels of influence (e.g. the organization, and/or individual). The PSC theory allows a multi-level perspective on work stress (Dollard, Opie et al., 2012).

The purpose of this study is to examine whether a complex intervention consisting of combined behavioral and organizational interventions for different target groups in hospital can reduce the work-related stressors of hospital staff and contribute to the improvement of their mental health and well-being.

# Trial Objectives and Purpose

This trial will evaluate the effect of a complex intervention on mental health and well-being of employees working in hospital.

## Primary objective

It will be assessed whether a complex intervention consisting of behavioural and structural preventive elements leads to an improvement in subjectively perceived emotional and cognitive strain of hospital employees measured with the irritation scale compared to standard care.

## Secondary objectives

The trial will also evaluate the effect of the complex intervention on the perceived emotional and cognitive strain in the working environment, subjective psychological well-being, the improved psychosocial safety climate, the perceived occupational effort as well as the perceived occupational rewards, the perceptions of working conditions, leadership and communication style and organizational indicators on individual/participant level as well as on hospital/clinic level. In this context, cluster participants not partaking in the modules will also be asked about their perception in order to evaluate a possible influence of the complex intervention on the organisation level.

## Process evaluation

Furthermore, a thorough process evaluation will be part of this trial. Up to four expert interviews will be conducted with persons not involved in the study to prepare the development of the interviews guidelines. In line with the best available evidence (Moore et al., 2015), a mixed-methods approach will be used. First, focus groups (Richter et al., 1991) (about N = 8 per group) will be conducted with a subsample of participants from all study sites before and after the implementation of the intervention to identify participants’ attitudes, perceptions, and experiences regarding the intervention. Three pre-post focus groups will be held at each study site, with participants representing an adequate mix of targets groups. If new and interesting topics relevant for the process evaluation are coming up during the interviews, additional interviews via video conferences will be conducted with participants from different study sites. The interviews will be analysed using qualitative content analysis (Mayring, 2015) via MAXQDA after transcription. Second, the entire aggregated data set will be subjected to comprehensive multivariate analysis using multilevel modelling to identify mediators and moderators of effect with a focus on context (e.g. organizational indicators), intervention (e.g. fidelity), participant characteristics, process measures (e.g. self-efficacy), and satisfaction ratings. Process evaluation includes the development of a pragmatic SEEGEN fidelity scale. The fidelity scale aims to assess and monitor fidelity of the complex process of intervention implementation in SEEGEN. Development of the scale and item selection was guided by recommendations of relevant methodological literature (Breitenstein et al., 2010; Carroll et al., 2007; Feely et al., 2018; Ibrahim & Sidani, 2015). Operational fidelity as the degree to which the intervention is delivered according to the original plan was a leading feature. A consensus-based selection of the final items was performed with a systematic rating within the study group. Five specific dimensions of fidelity are addressed:

1. Exposition (dose, frequency and duration of contact to the intervention)
2. Engagement (extent to which the participants are involved in the intervention activities and capture activities during the intervention sessions)
3. Enactment/adherence (extent to which participants actively apply the skills learned during the intervention in their daily routine)
4. Perceived competence and skills of the interventionist
5. Satisfaction with the intervention

The SEEGEN fidelity will be administered to recipients of the intervention alongside the SEEGEN RCT.

# Outcomes

## Primary Outcome

The outcome will be the change in the total score of the IRR (Mohr et al., 2005) from baseline (T0) to 11 months after baseline (T2*). The eight-item IRR assesses the emotional and cognitive strain in the working environment. Items are measured on a 7-point Likert scale, ranging from 1 (not at all) to 7 (almost completely correct).

## Secondary Outcomes

### Main secondary outcomes

- Change in the World-Health Organization Well-Being Index (WHO-5) from T0 to T2*. The WHO-5 is described below.
- Change in Psychosocial Safety Climate (PSC-12) from T0 to T2*. The PSC-12 is described below.

### Further secondary outcomes

In addition to the primary outcome, the change in the total score of the IRR from baseline to 11 months after baseline (T1*) will be evaluated as well as the changes from T0 to T1* and from T0 to T2* for both subscales, respectively. Further secondary outcomes are the change from T0 to T2* and from T0 to T1* in:

- World Health Organization Well-Being Index (WHO-5 Well-Being Index, Topp et al., 2015)

Subjective psychological well-being will be measured using the five-item World Health Organization Well-Being Index (WHO-5). Items are measured on a 6-point Likert scale, ranging from 0 (at no time) to 5 (all of the time). The raw score ranging from 0 to 25 is multiplied by 4 to give the final score ranging from 0 representing the worst well-being to 100 representing the best well-being.

- Psychosocial Safety Climate (PSC-12)

Psychosocial safety climate will be measured using the 12-item Psychosocial Safety Climate Scale (G. B. Hall et al., 2010). The PSC-12 consists of four dimensions: (1) organization participation (3 items), (2) organization communication (3 items), (3) management priority (3 items) and (4) management commitment (3 items). Items are measured on a 5-point Likert scale, ranging from 1 (strongly disagree) to 5 (strongly agree).

- Effort-Reward Imbalance Scale – Short version (ERI; Siegrist et al., 2009).

Effort will be measured by three items (ERI1-ERI3). Items are measured on a 4-point Likert scale, ranging from 1 (strongly disagree) to 4 (strongly agree) with higher sum score between 3 and 12 indicating more perceived efforts. Reward is measured by seven items (ERI4-ERI10). A low sum score of these items between 7 and 28 represents fewer perceived occupational rewards.

- Work Analysis Instrument for Hospitals – Self-Report Version (Tätigkeits- und Arbeitsanalyseverfahren für das Krankenhaus; TAA-KH-S; Glaser, 1997)

Two scales of a German questionnaire for hospital employees will be used to measure their working conditions: (1) scope of action (shortened from 9 to 3 items); (2) temporal overload (3 items). Items are measured on a 5-point Likert scale, ranging from 1 (no, not at all) to 5 (yes, absolutely).

- Questionnaire on integrative leadership (FIF) (Rowold & Poethke, 2017)

The questionnaire on integrative leadership is a standardized instrument which records leadership and communication style in four modules (module A: transformational and transactional leadership, module B: instrumental leadership, module C: communication and module D: negative leadership). In this study, module A in its self-assessment for leaders and its external assessment for subordinates will be used. The multidimensional construct of transformational leadership consists of six core behaviors (“fostering innovation”, “team spirit development”, “performance development”, “individuality focus”, “providing a vision” and “being a role model”). Participants have to rate 24 statements at a five point Likert-scale from 1 “agree not at all” to 5 “totally agree”. The items` ratings can be summarized to 10 different scale scores or to one overall score each (transformational leadership, transactional leadership and negative leadership). The scales of transformational leadership show a sufficient intern consistency with Cronbach`s α = .79 – .92 for the external assessment and Cronbach`s α = .71 – .83 for the self-assessment (Rowold & Poethke, 2017).

- Short version of the Occupational Self-Efficacy Scale (SOSES; Rigotti et al., 2008)

Occupational self-efficacy will be measured using the Short version of the Occupational Self-Efficacy Scale. The instrument consists of 6 items rated on a six-point Likert scale ranging from 1 (not at all true) to 6 (completely true) with higher values reflecting higher occupational self-efficacy.

- Top Management Evaluation Sheet

Participants in the top management will be asked to complete an evaluation form consisting of 29 items. The questions relate to measures to promote the mental health of employees at the hospital (e.g. offers for stress prevention, activities to improve working conditions) and the role of the management in this issue.

- Organizational indicators on individual/participant level:
  1. Job satisfaction (Fischer & Lück, 2001; 8 items, measured on different 5-point Likert scales, ranging from 1 “wrong” to 5 “right”, 1 “not at all interesting” to 5 “yes, very interesting”, 1 “very view opportunities” to 5 “yes, a lot of opportunities”, 1 “very discontent” to 5 “yes, very content”, 1 “definitely not” to 5 “yes, for sure”)
  2. Employer attractiveness (Bruhn et al., 2013; 5 items, measured on a 7-point Likert scale, ranging from 1 “strongly disagree” to 7 “strongly agree”)
  3. Presenteeism (Preisendörfer, 2010; 1 item, measured on a 5-point scale; “never”, “seldom”, “occasionally”, “frequently”, “very often”)
  4. Intention to leave (Simon et al., 2005; 1 item, measured on a 5-point scale; “never”, “a few times a year”, “a few times a month”, “a few times a week”, “everyday”)
  5. Absenteeism (modified version of Caverley et al., 2007; 1 item, “Please estimate, how many days on average per month you are being absent due to an illness.”)
  6. Recommendation (modified version of Reichheld, 2003; 1 item, measured on an 11-point Likert scale, ranging from 1 “highly unlikely” to 11 “highly likely”)
  7. Work overtime (“How many hours have you worked overtime in the past month?”; “What is your contracted duration of working time per week (in hours)?”)
  8. Economic situation of clinic/unit (adapted version of A. Hall & Rohrbach-Schmidt, 2013, “How do you assess the economic situation of the clinic/unit you work at?”, measured on a 5-point Likert scale, ranging from 1 “very good” to 5 “don’t know”).
  9. Job security (Lee et al., 2004, “My job security is good”, measured on a 4-point Likert scale, ranging from 1 “strongly disagree” to 4 “strongly agree”).
  10. Cooperation between occupational groups (Glaser, 1997, shortened from 5 to 2 items, TAA-KH-S subscale social climate, measured on a 5-point Likert scale, ranging from 1 “no, not at all” to 5 “yes, absolutely”).
- Exposure and (mental) effects caused by COVID-19 (12 items and five open questions for example on the extend of (mental) burden, actions by the employer and coping with the pandemic)

Additional secondary outcomes will be colltected at T2 only:

- Personal data that provide information about the number of own children, childcare facilities and the partner's work activity (9 items),
- Trierer Scale to measure Work-Life Balance (TKS-WLB, Syrek et al., 2011)

Work-Life Balance will be measured using the Trierer Scale to measure Work-Life Balance. The instrument consists of 5 items rated on a six-point Likert scale ranging from 1 (strongly disagree) to 6 (strongly agree).

- Copenhagen Psychosocial Questionnaire

The subscales “emotional demands” and “hiding emotions” of the Copenhagen Psychosocial Questionaaire will be used to measure emotional demands at work (COPSOQ , 4 items Nübling, 2005). Items are measured on a 5-point Likert scale, ranging from 1 (to a very large extent) to 5 (to a very small extent).

Moreover, some organizational indicators on hospital/clinic level will be collected annually via request from the hospitals accounting division and will be assigned to the clusters, if possible, depending on the level of detail of the data and the homogeneity/heterogeneity of the clusters.

- 1. Turnover ($\text{turnover rate = }\frac{\text{number of employees who left in year x}}{\text{total number of employees at the beginning of year x}}\text{ x 100}$,

number of applicants, number of persons hired, number of persons retired, number of dismissals, number of expiring fixed-term contracts)

- 1. $\text{Absence rate = }\frac{\text{absence (in hours) in year x}}{\text{gross working time (number of employees * working hours per week) in year x}}\text{ x 100}$
  2. Work overtime rate = $\frac{\text{work overtime (in hours) in year x}}{\text{contracted working time }\text{(}\text{in hours}\text{)}\text{ in year x}}\text{ x 100}$
  3. $\text{Sickness} \text{absence rate = }\frac{\text{sickness absence }\text{(}\text{in hours}\text{)}\text{ in year x}}{\text{planned working time }\text{(}\text{in hours}\text{)}\text{ in year x}}\text{ x 100}$
  4. Age distribution = number of employees sorted by age classes

In addition, participants of a cluster who decide not to participate in the modules (cluster participants) will be asked to complete a shorter questionnaire at T0, T1* and T2* consisting of the following measurements:

- - - IRR (Mohr et al., 2005), as described above,
    - WHO-5 (Topp et al., 2015), as described above and
    - PSC-12 (G. B. Hall et al., 2010), as described above,
    - GTL (Carless et al., 2000) scale to measure transformational leadership and
    - COVID-19 measures, as describes above.

Based on single items, they will be asked about their perception of the needs and benefits (personal and for the units they work at) of the module-offer, too. The outcome will be the change in the scores of IRR, WHO-5, PSC-12, GTL and single items from T0 to T2*, as well as from T0 to T1*.

## Process measures

- Context: Characteristics of cluster (hospital unit): total number of employees, employees participating from cluster, characteristics of employees (age, gender, job position).
- Intervention: module I-V preferences, dose (overall number of sessions, sessions in module I-V), UPSIDES fidelity scale (see 5.3).

# Modules of the complex intervention

The goal of the complex intervention is to improve managing work-related challenges in order to strengthen the well-being (physical and mental health) of hospital employees at different levels of hierarchy and functional areas. The complex intervention is built on experiences from five previous pilot projects carried out between 2017-19. These prior pilots operate on both, the behavioral and the structural levels, and will be used as a combined stress mitigating additive during the complex 10-month intervention period in three locations including clusters from small regional hospitals to university hospitals with full clinical care. A total of five individual modules with different thematic priorities will be offered: (i) Top Management Training, (ii) Promoting Stress preventive relational leadership competence) (iii) Dilemma Competency – Coping by taking responsibility, (iv) Reconciling Work and Family Life and (v) Stay Healthy at work. Importantly and in contrast to other studies, employees may choose one module according to their specific needs and interests depending on their position in the hospital. The maximum duration of one module shall be 12 hours.

## Top Management Training

The purpose of this intervention offered dealing with this subject is to raise awareness and competence among executives regarding the design of health-promoting work and its economic effects. The module to raise awareness includes measures to sensitize management staff on the departmental level (i.e. the leaders of the cluster) for issues of mental health of their employees. Moreover the module includes practical guidance to psychosocial health risk assessment and the development of structures as well as procedures to improve work conditions in the round tables. It is followed by a phase in which participants try to implement the required structures and procedures into occupational practice. The results of the transfer will be discussed in a follow up meeting on a collegial level in order to support further approaches.

## Promoting stress preventive relational leadership competence

Leadership behavior has been described as an important variable in relation to employees` psychological well-being (e.g. Montano et al., 2017). Since the hospital can be perceived as a mental health demanding workplace for employees (e.g., manpower shortage, economical pressure) it is the leader`s responsibility to preserve and foster subordinates` mental health. Therefore, we provide a leadership intervention which concentrates on relational and stress preventive aspects of leadership. It is open for middle management of all occupational groups at hospital and consists of four modules with a total duration of twelve hours. Between two modules a practice phase during which participants implement their new knowledge and techniques into their situation at work is conducted. Every module comprises theoretical parts (e.g., theoretical input through frontal presentations, instructional videos) to generate new knowledge as well as practical and interactive parts (e.g., group discussions, role pays, single work or partner work) to discuss and transfer this knowledge into the everyday work. Concerning the content the modules focus on a) a competent handling of leaders` stress as a requirement for leading, b) health and relational oriented leadership behavior like transformational leadership (Podsakoff et al., 1990) and Leader – Member-Exchange (LMX; Graen & Uhl-Bien, 1995) to provide successful working relations, c) motives, needs and stressors of subordinates to foster a mindful communication and d) managing team meetings and understanding team dynamics to lead teams stress preventive.

## Dilemma Competency – Coping by taking responsibility

For this intervention, an already successfully implemented training (Zwack & Bossmann, 2017) on dilemma competency to meet the working conditions in the public health sector has been adapted. Each group intervention consists of 2 days of 6 hours each and is administered over a period of 2 months.

The aim of the intervention is to provide the participants with mental and action-related abilities to understand contradictory demands in their organization and to meet associated demands constructively through negotiation and decision-making processes.

Overall the element of the complex intervention strives for prevention of stress-related diseases and the promotion of an enhanced sense of meaningfulness experienced by the participants. In times of an increasing aggravation of working conditions due to economic strain, competitive constraints and shortage of skilled employees in the health care system, dilemma management supports employees in dealing with daily work strains.

## Reconciling Work and Family Life

The one-day module lasts six hours (four modules 90 minutes each) and the maximum number of participants is 15. Aim of the module is to improve one's ability to cope with reconcilability stress by enabling a personal analysis and reflection of each participant's situation. This analysis of the professional and family environment is guided by worksheets specifically developed for the intervention, which are worked on individually or in groups. Further central elements are the subject-related impulse lectures regarding the connection between stress experience, stress response and the influence of personal stress on the relationship to the child as well as developmental psychological findings for practical everyday life as a parent. The participants are also instructed on practical stress management in the form of yoga. In contrast to a pure coping workshop, this module is also highlighting the constellation "dilemma" in the context of a role-play, in order to work out how to deal with an unsatisfactorily solvable compatibility situation in a further step.

## Stay Healthy at Work

In a small group intervention with up to 15 participants on two days the topics age and getting older at work, reflection on one's professional biography and defining resources, dealing with stressors, Selection, Optimization and Compensation (SOC) concept, personal processing of an individual project within the SOC framework, physical health and work-life balance will be dealt with. The participants will be informed about various coping strategies such as: relaxation exercises/techniques (autogenous training, fantasy trips, PMR) and mindfulness exercises.

## Roundtable discussions and booster sessions

In addition to the modules, roundtable discussions were offered to facilitate organizational change and develop concrete proposals for action. Due to the small number of participants, the following adjustments were made to the round tables: Instead of one round table per module type, one round table together with one booster session were offered for all module types at each study location. Booster sessions were offered to enhance training effects. In these booster sessions the exchange of transfer experiences from training into daily hospital life is enabled. It is hoped that some participants will continue to exchange experiences in a self-organizing fashion, even after project termination. The duration for booster sessions and 1^st^ order round tables is 3-4 hours.

The Round Tables are a participation instrument in which representatives of different positions and interests come together under a neutral moderation in order to process various suggestions for improvement made by employees during the modules. As a first step, concrete measures operating on the structural level will be developed (so-called 1^st^-order round tables), which will be presented in a second step to the decision-makers at each study location (including the Executive Board; so-called 2^nd^-order round tables). The duration of the 2^nd^-order round tables is 3-4 hours.

# Design of the Trial

## Trial Design

The study is designed as a multi-centre cluster-randomized open trial with a waitlist control group.

## Randomisation and Blinding

For organizational reasons, cluster-randomization will be carried out prior to recruitment of the first participants. The allocation will be done stratified by the three locations in a 1:1 fashion using a randomization list which will be prepared by the Institute of Medical Biometry and Informatics. If appropriate, cluster pairs per site will be defined in order to prevent that matched pairs will be randomized into the same group. Due to the nature of the intervention blocks, blinding of participants and trainers is not possible.

## Additional Methods for Reducing Bias

Participants will be given access to a separate room to fill out the questionnaires.

## Duration of Trial Period(s)

Total trial duration is 12 months, consisting of a 10-month intervention phase. The different intervention modules vary in their duration: The first intervention module – Top Management Training– has a duration of one working day, the Promoting stress preventive relational leadership competence has a total duration of 3 weeks. The third intervention module – Dilemma Competency – Coping by taking responsibility – will be administered over a period of 2 months. The intervention module Reconciling Work and Family Life has a duration of one working day while Stay Healthy at Work is offered over a period of 4 weeks. Booster sessions and the 1^st^-order round tables will take place at the beginning of October 2020, the 2^nd^-order round tables will be held at the end of October, before T2*.

# Study population and withdrawal of subjects

## Number of Subjects

All employees in the participating units of the three study locations (Aalen/Ellwangen, Heidelberg, Duisburg) will be potential participants. At least six clusters are planned per location, with altogether in about 720 potential participants (360 participants in intervention and 360 participants in waitlist control group).

## Subject Inclusion Criteria

Inclusion criteria for study locations are:

- Willingness to participate in the study regardless of randomization to the intervention or waitlist control arm
- Willingness to complete three questionnaires (applicable to intervention group, waitlist control group and cluster participants)

Eligible criteria for employees who want to take part in one or more interventions are:

1. Age: 18-70 years old,
2. Written informed consent,
3. Sufficient German language skills to fill out the questionnaires

## Subject Exclusion Criteria

Exclusion is limited to the negotiation of the inclusion criteria.

## Subject Withdrawal Criteria

All participants are informed that they can terminate their participation in the study at any time without stating reasons.

# Study process

Eligible clusters will be identified in three study locations (Aalen/Ellwangen, Heidelberg, Duisburg). Interested employees in these clusters will be informed about the study and the different interventions by means of information events or by the operational health management of the respective location. Verbal and written information will be given to all employees interested in the interventions. The study staff will contact potential participants and obtain written informed consent. A choice of five modules will be offered to the study participants with the restrictions mentioned above. For participants in clusters randomized into the intervention group, the remaining modules will start within the intervention phase. For participants in clusters randomized into the waitlist control group, the five modules will begin after the intervention phase. As a possible disadvantage, the participants in the waitlist control group might feel deterred by the long waiting time of 12 months.

# Safety

The collected data will comprise of self-reported questionnaires, personal interviews and process produces data e.g. from modules. The intervention and control group will not undergo any invasive or potentially harmful procedure, therefore no adverse events or safety parameter definition is necessary.

# Data Collection, Data Management and Archiving

## Data collection

Individual participant data (demographics and questionnaires on individual level) will be collected at T0, T1* and T2* (starting end of 2019) either paper based (CRF) or web based electronically (eCRF) in order to meet individual preferences.

Organisational indicators (on hospital/clinic level, assigned to clusters) will be collected either paper based or electronically as well. Paper based questionnaires will be sent to the IMBI by post. Data will be captured via double data entry near term to receipt and it will be transferred to a statistical analysis system running on a server located in the IMBI subsequently to data capture.

Data captured in the eCRF will be transferred to the analysis system as well. The electronic data capture system (EDC-system) underlying the eCRF is also running on a server located in the IMBI. Data related to process evaluation will be collected and stored separately.

## Data Management

The system used for web based electronic data capture (EDC-system) is validated and compliant with FDA 21 CRF part 11. Data transmission is encrypted with secure socket layer (SSL) technology. The database server in the IMBI is located in a secure data centre and protected by a firewall. The system provides an infrastructure to support user roles and rights.

Only authorized users are able to enter or edit data. The access to centre specific organisational data is restricted to persons of the respective centre. Access to individual data is restricted to the participant only. All changes to data are logged with a computerized timestamp in an audit trail within the EDC-system.

Any modification of data captured from paper based questionnaires via double data entry (e.g. as result of queries) will be logged within the analysis system as well.

All individual data will be captured, transferred and stored in a pseudonymized manner.

Backups of both, EDC-system and the statistical analysis system are conducted regularly.

High quality of data will be guaranteed by checking for completeness, consistency and plausibility on or near term to data entry. This will be realized by implementation of programed validation rules, predefined in a data validation plan. The validation programs will generate queries/edit checks on data entry within the EDC-System and/or subsequent to data transfer to the analysis system by generating paper based queries. The investigators or the designated representatives are obliged to clarify or explain the edit-checks and queries.

During study conduct, the database is accessible to the data manager and data entry staff only. After database closure, access rights will be granted to the responsible biometricians as well.

Data will be managed and analysed according to the corresponding Standard Operating Procedures (SOPs) valid in the IMBI.

Business data will be transferred to the Chair of Business Administration, esp. Organization and Human Resources of the Heinrich-Heine-University Düsseldorf for analyses.

## Archiving

Study data and study documents stored in the IMBI will be transferred to the study coordinator subsequent to statistical report and/or publication for archiving.

# Statistics

## Sample Size Calculation

The sample size calculation is based on the primary endpoint, i.e. the absolute change in the total score of the IRR from T0 to T2*, which will be evaluated by an ANCOVA approach. The sample size is calculated based on a two-sided two sample t-test with two-sided significance level α = 0.05 and power 1-β = 0.8, which provides a conservative estimate for the necessary sample size for the ANCOVA evaluation. The intervention is assumed to yield a medium effect, hence an effect size of d = 0.4 is used for sample size calculation. These assumptions yield a sample size of 100 participants per group (200 for both intervention and control). Taking into account the dependencies within clusters and simultaneously being conservative via assuming a high intraclass correlation coefficient of ICC = 0.05 and a mean cluster size of about 40 participants yields a design effect of 1 + 0.05*(40-1) = 2.95. Therefore, the necessary sample size is 2.95*200 $\approx$ 590 participants. Furthermore, a drop-out rate of 18% will be considered. Taken together, this amounts to a total sample size of 720 participants in 18 clusters. Under consideration of possible cluster drop-outs, more than 18 clusters might be recruited which will lead to an increased power. The sample size was calculated using the software SAS 9.4.

## Statistical Methods

The primary analysis tests the null hypothesis that the absolute change in the total score of the IRR from T0 to T2* in the intervention group is equal to the absolute change in the total score in the control group. The analysis of the primary endpoint will be based on an ANCOVA model comparing the treatment groups including the baseline value of the total score of the IRR at T0 (baseline) with the total score of the IRR at T2* (11 months after T0; follow-up) between intervention and control group, including hierarchy level (top management, middle management and employees without management responsibility) and gender (female, male) as well as the site (planned are three) as covariates and the respective cluster as random effect. In case of significance, the WHO-5 will be tested in hierarchical manner in the same way. Again, in case of significance, the PSC-12 will be tested. Applying this hierarchical testing strategy the overall type I error will be controlled. All other analyses will be of exploratory nature and interpreted only in descriptive manner. The primary analysis will be conducted based on the full analysis set reflecting the intention to treat principle including all participants in the group the respective cluster was randomized to. As sensitivity analyses, the per-protocol population (only participants which fulfil the inclusion criteria) will be analysed. There, only participants of the intervention group who participated in at least one session of a module are considered in the intervention group. Otherwise, these participants will be excluded from the analysis. The participation of individuals from the intervention group in further programs (round tables and booster sessions) will be considered as additional covariate in further analyses of primary and secondary endpoints.

The analysis of the secondary endpoints within the population of cluster participants will be based on an ANCOVA model as well, comparing the cluster participants including the baseline value of the total score of the IRR, WHO-5, PSC-12 or single items at T0, respectively, with the total score of the IRR, WHO-5, PSC-12 or single items at T2*, respectively, between cluster participants of a cluster randomized into intervention group and cluster participants of a cluster randomized into control group, including hierarchy level (top management, middle management, employees without management responsibility) and gender (female, male) as well as the site (planned are three) as covariates and the respective cluster as random effect. As additional secondary analyses, changes in the collected scores from T0 to T1* will be analysed.

All data of clusters and participants will be described by treatment group. Categorical data will be presented as frequencies and percentages. For continuous data, the number, mean, standard deviation, median, inter-quartile range, minimum and maximum will be calculated.

For further evaluation of potential factors influencing treatment effect, moderator and mediation analyses as well as subgroup analyses will be conducted. As example, the subgroup of a specific level of hierarchy will be evaluated. A detailed description of the planned statistical analyses will be provided in the Statistical Analysis Plan (SAP) which will be finalized prior to database closure and any analysis. All analyses will be conducted using SAS 9.4 or higher.

## Handling of Missing and Spurious Data and Drop outs

If the IRR, WHO-5 or PSC-12 is missing at T0 or T2* for a participant due to loss to follow-up or other reasons, analyses will be done replacing the missing follow-up value on item level with the predictive mean matching method. As sensitivity analysis, the baseline value will be carried forward resulting in no change for the primary endpoint. In addition, further sensitivity analysis will be conducted to evaluate the robustness of the results. If the complete IRR, WHO-5 or PSC-12 is missing at T0 and T2*, the participants will be excluded for primary analyses according to a modified intention to treat principle.

# Ethical and Legal Aspects

The trial will be conducted according to the Declaration of Helsinki.

## Subject Information and Informed Consent

Interested employees will be informed about the study and the different interventions by means of information events or by the operational health management of the respective location. Verbal and written information will be given to all employees interested in the interventions.

## Confidentiality

To maintain confidentiality, a pseudonomized identification coded number and year of birth only will identify all evaluation forms, reports and other records. All study records will be kept in a locked file cabinet and code sheets linking a patient’s name to a patient identification number will be stored separately in another locked file cabinet.

The storage, evaluation and transfer of study-related data is carried out in accordance with statutory provisions and requires the participant's voluntary written informed consent before participating in the study. The participants agree that data collected may be recorded on questionnaires and electronic data carriers and processed without naming. In addition, the participants agree that an authorised person who is bound to secrecy (e.g. persons conducting audits) may inspect the personal data collected insofar as this is necessary for the review of the project.

## Protocol Amendments

-

## Approval of the Clinical Trial Protocol and Amendments

Before enrolling the first subject in the trial, all appropriate documents will be submitted to the independent ethics committees.

## Ongoing Information of Independent Ethics Committee (IEC)

Not applicable

## Liability and Insurance

During the participation in the research project the participants have insurance cover. The University Hospital Ulm and its staff involved in the study (study physicians, other staff) are insured against liability in the event that the participants suffer damage through their fault.

# Declarations

This study protocol was subject to critical review. The information it contains is consistent with the current risk-benefit evaluation of the investigational product and the moral, ethical, and scientific principles governing clinical research as set out in the Declaration of Helsinki and the principles of ICH-GCP.

Date: _____30.10.20_____ Signature: _______________________________________

Name: Harald Gündel

Function: Coordinating Investigator / LKP

Date: _____30.10.20_____ Signature: _______________________________________

Name: Regina Krisam

Function: Biometrician

Date: ___ 30.10.20_____ Signature: _______________________________________

Name: Nadine Mulfinger

Function: Trial Coordinator

#

References

Agerbo, E., Gunnell, D., Bonde, J. P., Mortensen, P. B., & Nordentoft, M. (2007). Suicide and occupation: The impact of socio-economic, demographic and psychiatric differences. *Psychological Medicine*, *37*(8), 1131–1140.

Breitenstein, S. M., Gross, D., Garvey, C. A., Hill, C., Fogg, L., & Resnick, B. (2010). Implementation fidelity in community-based interventions. *Research in Nursing & Health*, *33*(2), 164–173.

Bruhn, M., Batt, V., & Flückiger, B. (2013). Aufbau von Arbeitgeberattraktivität – Identifikation der Determinanten und empirische Überprüfung. *Die Unternehmung*, *67*(1), 62–82.

Carless, S. A., Wearing, A. J., & Mann, L. (2000). A short measure of transformational leadership. *Journal of Business and Psychology*, *14*(3), 389–405.

Carroll, C., Patterson, M., Wood, S., Booth, A., Rick, J., & Balain, S. (2007). A conceptual framework for implementation fidelity. *Implementation Science : IS*, *2*, 40.

Caverley, N., Cunningham, J. B., & MacGregor, J. N. (2007). Sickness Presenteeism, Sickness Absenteeism, and Health Following Restructuring in a Public Service Organization. *Journal of Management Studies*, *44*, 304–319.

Dollard, M. F. (2012). Psychosocial safety climate: A lead indicator of workplace psychological health and engagement and a precursor to intervention success. In *Improving organizational interventions for stress and well-being: Addressing process and context* (pp. 77–101). New York: Routledge/Taylor & Francis Group.

Dollard, M. F., & Bakker, A. B. (2010). Psychosocial safety climate as a precursor to conducive work environments, psychological health problems, and employee engagement. *Journal of Occupational and Organizational Psychology*, *83*(3), 579–599.

Dollard, M. F., LaMontagne, A. D., Caulfield, N., Blewett, V., & Shaw, A. (2007). Job stress in the Australian and international health and community services sector: A review of the literature. *International Journal of Stress Management*, *14*(4), 417–445.

Dollard, M. F., Opie, T., Lenthall, S., Wakerman, J., Knight, S., Dunn, S., . . . MacLeod, M. (2012). Psychosocial safety climate as an antecedent of work characteristics and psychological strain: A multilevel model. *Work & Stress*, *26*(4), 385–404.

Feely, M., Seay, K. D., Lanier, P., Auslander, W., & Kohl, P. L. (2018). Measuring Fidelity in Research Studies: A Field Guide to Developing a Comprehensive Fidelity Measurement System. *Child and Adolescent Social Work Journal*, *35*(2), 139–152.

Fischer, L., & Lück, H. E. (2001). *Allgemeine Arbeitszufriedenheit: Zusammenstellung sozialwissenschaftlicher Items und Skalen*. Retrieved from http://zis.gesis.org/pdfFiles/Dokumentation/Fischer+%20Allgemeine%20Arbeitszufriedenheit.pdf https://doi.org/10.6102/ZIS1

Glaser, J. (1997). *Aufgabenanalysen in der Krankenpflege. Eine psychologische Analyse und Bewertung pflegerischer Aufgaben [Task analysis in nursing. A psychological analysis and evaluation of nursing tasks]*. Münster: Waxmann.

Graen, G. B., & Uhl-Bien, M. (1995). Relationship-based approach to leadership: Development of leader-member exchange (LMX) theory of leadership over 25 years: Applying a multi-level multi-domain perspective. *The Leadership Quarterly*, *6*(2), 219–247.

Hall, A., & Rohrbach-Schmidt, D. (2013). *BIBB/BAuA employment survey 2012. BIBB-FDZ Data and Methodological Reports*. Bonn, Germany: Federal Institute for Vocational Education and Training.

Hall, G. B., Dollard, M. F., & Coward, J. (2010). Psychosocial Safety Climate: Development of the PSC-12. *International Journal of Stress Management*, *17*(4), 353–383.

Ibrahim, S., & Sidani, S. (2015). Fidelity of Intervention Implementation: A Review of Instruments. *Health*, *07*(12), 1687–1695.

Lee, S., Colditz, G. A., Berkman, L. F., & Kawachi, I. (2004). Prospective study of job insecurity and coronary heart disease in US women. *Annals of Epidemiology*, *14*(1), 24–30.

Mathieu, J. E., & Taylor, S. R. (2007). A framework for testing meso-mediational relationships in Organizational Behavior. *Journal of Organizational Behavior*, *28*(2), 141–172.

Mayring, P. (2015). *Qualitative Inhaltsanalyse. Grundlagen und Techniken*. Weinheim, Basel: Beltz.

Mohr, G., Rigotti, T., & Müller, A. (2005). Irritation - ein Instrument zur Erfassung psychischer Beanspruchung im Arbeitskontext. Skalen- und Itemparameter aus 15 Studien [Irritation - an instrument assessing mental strain in working contexts. Scale and item parameters from 15 studies]. *Zeitschrift für Arbeits- und Organisationspsychologie A&O*, *49*(1), 44–48.

Montano, D., Reeske, A., Franke, F., & Hüffmeier, J. (2017). Leadership, followers' mental health and job performance in organizations: A comprehensive meta-analysis from an occupational health perspective. *Journal of Organizational Behavior*, *38*(3), 327–350.

Moore, G. F., Audrey, S., Barker, M., Bond, L., Bonell, C., Hardeman, W., . . . Baird, J. (2015). Process evaluation of complex interventions: Medical Research Council guidance. *BMJ (Clinical Research Ed.)*, *350*, h1258.

Nübling, M. (2005). *Methoden zur Erfassung psychischer Belastungen: Erprobung eines Messinstrumentes (COPSOQ) ; [Abschlussbericht zum Projekt "Methoden zur Erfassung psychischer Belastungen - Erprobung eines Messinstrumentes (COPSOQ)" - Projekt F 1885*. *Schriftenreihe der Bundesanstalt für Arbeitsschutz und Arbeitsmedizin Forschung: Vol. 1058*. Bremerhaven: Wirtschaftsverl. NW Verl. für Neue Wiss.

Podsakoff, P. M., MacKenzie, S. B., Moorman, R. H., & Fetter, R. (1990). Transformational leader behaviors and their effects on followers' trust in leader, satisfaction, and organizational citizenship behaviors. *The Leadership Quarterly*, *1*(2), 107–142.

Preisendörfer, P. (2010). Präsentismus. Prävalenz und Bestimmungsfaktoren unterlassener Krankmeldungen bei der Arbeit. *German Journal of Human Resource Management: Zeitschrift für Personalforschung*, *24*(4), 401–408.

Reichheld, F. E. (2003). The One Number You Need to Grow. *Harvard Business Review*, *81*(12), 46–54.

Richter, J. M., Bottenberg, D. J., & Roberto, K. A. (1991). Focus group: Implications for program evaluation of mental health services. *Journal of Mental Health Administration*, *18*(2), 148–153.

Rigotti, T., Schyns, B., & Mohr, G. (2008). A Short Version of the Occupational Self-Efficacy Scale: Structural and Construct Validity Across Five Countries. *Journal of Career Assessment*, *16*(2), 238–255.

Rowold, J., & Poethke, U. (2017). *Fragebogen zur integrativen Führung (FIF)*. Bern: Hogrefe.

Ruotsalainen, J. H., Verbeek, J. H., Mariné, A., & Serra, C. (2015). Preventing occupational stress in healthcare workers. *The Cochrane Database of Systematic Reviews*. (4), CD002892.

Siegrist, J., Wege, N., Pühlhofer, F., & Wahrendorf, M. (2009). A short generic measure of work stress in the era of globalization: Effort-reward imbalance. *International Archives of Occupational and Environmental Health*, *82*(8), 1005–1013.

Simon, M., Tackenberg, P., Hasselhorn, H.‑M., Kümmerling, A., Büscher, A., & Müller, B. H. (2005). *Auswertung der ersten Befragung der NEXT-Studie in Deutschland*. Universität Wuppertal. Retrieved from http://www.next.uni-wuppertal.de

Syrek, C., Bauer-Emmel, C., Antoni, C., & Klusemann, J. (2011). Entwicklung und Validierung der Trierer Kurzskala zur Messung von Work-Life Balance (TKS-WLB). *Diagnostica*, *57*(3), 134–145.

Tetrick, L. E., & Winslow, C. J. (2015). Workplace Stress Management Interventions and Health Promotion. *Annual Review of Organizational Psychology and Organizational Behavior*, *2*(1), 583–603.

Topp, C. W., Østergaard, S. D., Søndergaard, S., & Bech, P. (2015). The WHO-5 Well-Being Index: A systematic review of the literature. *Psychotherapy and Psychosomatics*, *84*(3), 167–176.

Van Wyk, B. E., & Pillay-Van Wyk, V. (2010). Preventive staff-support interventions for health workers. *The Cochrane Database of Systematic Reviews*. (3), CD003541.

Zohar, D. (2010). Thirty years of safety climate research: Reflections and future directions. *Accident; Analysis and Prevention*, *42*(5), 1517–1522.

Zwack, J., & Bossmann, U. (2017). *Wege aus beruflichen Zwickmühlen: Navigieren im Dilemma*. Gottingen: Vandenhoeck & Ruprecht. Retrieved from http://search.ebscohost.com/login.aspx?direct=true&scope=site&db=nlebk&AN=1617322
