# Supplementary material for: Effectiveness of a multilevel intervention to improve mental health of hospital workers: The SEEGEN multicenter cluster randomized controlled trial
Source: PLoS One. 2025 Aug 21;20(8):e0330490. doi: 10.1371/journal.pone.0330490 (PMC12370060; doi:10.1371/journal.pone.0330490)
Supplement: S2 Document — (DOCX) [file pone.0330490.s004.docx]

**Universität Ulm Nr. / Jahr**

# Ethikkommission

Darstellung von Forschungsprojekten über wissenschaftliche Untersuchungen am Menschen

1. Titel des Projektes:

Seelische Gesundheit am Arbeitsplatz Krankenhaus (SEEGEN)

Studienleitung^[[1]](#footnote-1)^: Prof. Dr. med. Harald Gündel

Zahl der in die Studie aufgenommenen Patienten/Probanden: N = 720

1. Quantitative clusterrandomisierte Studie:

Drei Studienzentren, insgesamt 18 Cluster (d.h. 6 pro Studienzentrum)

- Ostalbkliniken Aalen (6 Cluster mit 40 teilnehmenden Mitarbeitern) n = 240
- Universitätsklinikum Heidelberg (6 Cluster mit 40 teilnehmenden Mitarbeitern)

n = 240

- Helios Klinikum Duisburg (6 Cluster mit 40 teilnehmenden Mitarbeitern)

n = 240

1. qualitative Fokusgruppeninterviews – Prozessevaluation (N = 32)

x Es handelt sich um eine prospektive Untersuchung.

Es handelt sich um eine retrospektive Untersuchung.

x Die Daten werden pseudonymisiert verarbeitet.

Die Daten werden nach Abschluss der Untersuchung vollständig anonymisiert,

eine Zuordnung zu einzelnen Personen ist dann nicht mehr möglich.

**2.** Projektleiter:

Prof. Dr. med. Harald Gündel, Ärztlicher Direktor der Klinik für Psychosomatische Medizin und Psychotherapie, Universitätsklinikum Ulm

a) Ärztliche Kooperationspartner / Mitarbeiter:

- - Prof. Dr. med. Peter Angerer, Institut für Arbeits-, Sozial- und Umweltmedizin. Universitätsklinikum Düsseldorf
  - Prof. Dr. med. Stephan Zipfel, Prof. Dr. med. Monika Rieger und Dr. med. Florian Junne, Klinik für Psychosomatische Medizin, Universitätsklinikum Tübingen
  - Dr. med. Eva Rothermund, Klinik für Psychosomatische Medizin, Universitätsklinikum Ulm
  - Dr. med. Imad Maatouk, Klinik für Psychosomatische Medizin, Universitätsklinikum Heidelberg

b) Nichtärztliche Kooperationspartner / Mitarbeiter:

- - Prof. Dr. phil. Andreas Müller und Melanie Genrich, Arbeits- und Organisationspsychologie, Universität Duisburg-Essen
  - Prof. Dr. rer.soc. Jochen Schweitzer-Rothers, Ulrike Bossmann, Dr. sc. hum. Julika Zwack, Marieke Born, Antonia Drews, Institut für Medizinische Psychologie, Universitätsklinikum Heidelberg
  - Felicitas Stuber und Elena Tsarouha, Klinik für Psychosomatische Medizin, Universitätsklinikum Tübingen
  - Prof. Dr. phil. Ute Ziegenhain, Manuela Gulde und Franziska Köhler-Dauner, Klinik für Kinder- und Jugendpsychiatrie, Universitätsklinikum Ulm
  - Dr. biol. hum. Lucia Jerg-Bretzke, Klinik für Psychosomatische Medizin, Sektion Medizinische Psychologie, Universitätsklinikum Ulm
  - Dr. biol. hum Nadine Mulfinger und Dr. Marc N. Jarczok, Klinik für Psychosomatische Medizin, Universitätsklinikum Ulm
  - Madeleine Helaß und Sebastian Götz, Klinik für Psychosomatische Medizin, Universitätsklinikum Heidelberg
  - PD Dr. Bernd Puschner, Maja Stiawa, Martin Peters, Klinik für Psychiatrie und Psychotherapie II der Universität Ulm
  - Prof. Dr. sc. hum. Meinhard Kieser, Dr. sc. hum. Anja Sander, Christina Klose, Regina Brinster und Ronald Limprecht, Institut für Medizinische Biometrie und Informatik, Abteilung Medizinische Biometrie der Universität Heidelberg
  - Prof. Dr. rer. pol. Stefan Süß, Dr. rer. oec. Sascha Ruhle und Elena Gesang, Lehrstuhl für BWL, insb. Organisation und Personal, Heinrich-Heine-Universität Düsseldorf
  - Dr. PH Karl Blum, Leiter Geschäftsbereich Forschung, Vorstand, Deutsches Krankenhausinstitut e.V.
  - Britta Worringer, Institut für Arbeits-, Sozial- und Umweltmedizin, Universitätsklinikum Düsseldorf

**3.** Ort der beabsichtigten Untersuchungen:

- Ostalbkliniken Aalen, Standorte Ellwangen und Aalen,
- Universitätsklinikum Heidelberg,
- Helios Klinikum, Standort Duisburg

**4.** Beschreibung des Forschungsprogrammes:

Im Rahmen des Forschungsprojektes wird untersucht, ob eine komplexe Intervention aus verhaltens- und verhältnispräventiven Elementen zu einer Verbesserung der subjektiv wahrgenommenen emotionalen und kognitiven Belastung der Krankenhausmitarbeiter führt, gemessen mit der Irritationsskala (Mohr et al. 2005) im Vergleich zur Standardversorgung. Die komplexe Intervention wird an drei Klinikstandorten implementiert und in einer clusterrandomisierten Studie evaluiert.

Die Studie wird auch die Auswirkungen der komplexen Intervention auf das subjektive psychische Wohlbefinden, das psychosoziale Sicherheitsklima, die wahrgenommene berufliche Leistung sowie die wahrgenommenen beruflichen Belohnungen, die gesundheitsbezogene Lebensqualität, die Wahrnehmung der Arbeitsbedingungen, die Emotionsregulationsstrategien, die soziale Unterstützung bei der Arbeit, den Führungs- und Kommunikationsstil, die Vereinbarkeit von Familie und Beruf und die organisatorischen Indikatoren auf individueller Ebene sowie auf Krankenhaus-/Klinikebene bewerten.

Das Hauptoutcome wird die Veränderung des Mittelwertes der Irritationsskala (Mohr et al. 2005) von Baseline (T0) zu 11 Monaten nach Baseline (T2*) sein. Die Irritationsskala (acht Items) bewertet die emotionale und kognitive Belastung im Arbeitsumfeld. Die Items werden auf einer 7-Punkte-Likert-Skala gemessen, die von 1 (überhaupt nicht) bis 7 (fast vollständig korrekt) reicht.

Alle Teilnehmer eines Clusters, die sich gegen die Teilnahme an einem Workshop entscheiden, werden gebeten, zu drei Messzeitpunkten einen gekürzten Fragebogen (s. Anhang) auszufüllen. Auf diese Weise können die Effekte des verhältnispräventiven Interventionsansatzes überprüft werden.

Die komplexe Intervention wurde anhand einer vorherigen zweijährigen Pilotphase entwickelt, in denen die einzelnen Interventionen erprobt und evaluiert wurden. Die einzelnen Interventionsbausteine werden im Folgenden näher beschrieben

1. Der Workshop – *Gestaltungskompetenz für gesunde Arbeit – lohnende, realisierbare Ansatzpunkte* – , welcher sechs Stunden dauert, hat zum Thema, die Kompetenz zur Gestaltung gesunder und motivierender Arbeitsbedingungen im Team zu fördern und Strukturen zur abteilungsübergreifenden Arbeit an organisationalen Verbesserungsmaßnahmen zu entwickeln. Der Workshop zur Sensibilisierung richtet sich an leitende Pflegekräfte und leitende Ärztinnen bzw. Ärzte die arbeitsorganisatorische Stressoren für das Team systematisch reduzieren möchten. In dem Workshop erfahren Führungskräfte, welchen erheblichen Einfluss Arbeitsbedingungen auf die Gesundheit und Leistungsfähigkeit ihrer Mitarbeitenden haben, welche 4 zentralen Schritte es zu berücksichtigen gilt, wenn es um eine gesunde Arbeitsgestaltung geht, wie sie interprofessionell und abteilungsübergreifend an ihrer Klinik konkrete organisationale Verbesserungsmaßnahmen entwickeln und umsetzen können.
2. Der Workshop *Dilemmakompetenz – schwierige Entscheidungen schaffen, ohne von ihnen geschafft zu werden* – richtet sich an Mitarbeiterinnen und Mitarbeiter aller Berufsgruppen, die häufig schwierige Entscheidungen treffen müssen und ihren Umgang mit solchen Entscheidungssituationen verbessern möchten. Das Training fördert die Entscheidungsfähigkeit in Situationen, in denen es keine zu hundert Prozent stimmige Lösung gibt, sondern jede Option mit Nachteilen verbunden ist. Das Training vermittelt u.a. ein Bewusstsein der eigenen Rolle in der Organisation Krankenhaus mitsamt den oft widersprüchlichen Anforderungen sowie der Zielkonflikte zwischen verschiedenen Abteilungen, ein Bewusstwerden der eigenen Entscheidungsspielregeln („Ich muss immer richtig entscheiden“) sowie der Organisationskultur. Das zweitägige Dilemmakompetenztraining findet an zwei Tagen mit vier bis acht Wochen Abstand in Gruppen von 10 bis 20 Teilnehmenden statt.
3. *Der Workshop stresspräventive Führungskompetenz: Ressourcen stärken durch teamorientierte Führung im Krankenhaus –* richtet sich an Führungskräfte der mittleren Führungsebene (z.B. Oberärzte/-innen, Pflegebereichsleitungen, Funktions-/Teamleitungen aller Berufsgruppen. Das Seminar bietet die Möglichkeit, Anregungen für den eigenen Umgang mit Stressbelastung und Drucksituationen kennenzulernen, die stresspräventive und mitarbeiterorientierte Führungskompetenz anhand innovativer Führungskonzepte zu erweitern, die eigenen Fertigkeiten im Bereich Kommunikation und Interaktion weiter zu entwickeln, um die Ressource „Team“ in komplexen Bezügen zu stärken. Das Seminar mit maximal 20 Teilnehmenden findet an zwei Tagen im Abstand von ungefähr drei Wochen statt.
4. In dem Workshop „Vereinbarkeit von Familie und Beruf“, welcher sechs Stunden dauert mit einer max. Teilnehmerzahl von 15, wurde speziell für Mitarbeiterinnen und Mitarbeiter im Pflege- und ärztlichen Dienst entwickelt, die sich in der Familienphase befinden. Ziel ist es, die Stresssituationen an der Nahtstelle zwischen Arbeit und Familie besser meistern zu können. Die Teilnehmenden des Workshops werden sensibilisiert, ihren individuellen Handlungsspielraum zu erweitern bzw. mögliche Stresssituationen zu erkennen, zu analysieren und zu entschärfen. Darum geht es u.a.: Analyse der eigenen familiären und beruflichen Situation, um Hindernisse und Ressourcen für eine gelungene Vereinbarkeit herauszuarbeiten, Vermittlung von Fakten über den Zusammenhang zwischen Stresserfahrung, Stressreaktion und dem Einfluss von persönlichem Stress auf die Beziehung zum Kind sowie entwicklungspsychologische Erkenntnisse für den praktischen Elternalltag, Erlernen von praktischen Übungen und Entlastungsstrategien (Atem- und Yogaübungen), die sich gut in den Alltag integrieren lassen.
5. *Gesund bleiben im Beruf – Erlernen von Strategien zur Selbstfürsorge und Ressorucenaktivierung*: Anhand dieser Gruppenintervention mit bis zu 15 Teilnehmern aller Berufsgruppen werden an zwei Terminen im Abstand von ca. 4 Wochen diverse Ansätze zur Bewältigung alters- und stressbedingter Veränderung mit Anleitung zur Umsetzung im Alltag behandelt. Das Training beinhaltet u.a. sowohl die gemeinsame Reflexion stress- und altersbedingter Veränderungen sowie den Austausch von Erfahrungen und persönlichem Erleben am Arbeitsplatz als auch die Vermittlung und Einübung von Methoden zur Verbesserung der Wahrnehmung von Belastungsanzeichen

Zusätzlich zu den Workshops finden Runde Tische statt, die organisationale Veränderungen ermöglichen und in denen konkrete Maßnahmenvorschläge erarbeitet werden. Pro Workshoptyp wird ein Runder Tisch stattfinden (ausgenommen hiervon ist der Workshop *Gestaltungskompetenz für gesunde Arbeit – lohnende, realisierbare Ansatzpunkte*), d.h. es werden jeweils für Interventions- und Kontrollgruppe vier Runde Tische an den jeweiligen Standorten stattfinden. Bei den Runden Tischen handelt es sich um ein Beteiligungsinstrument, an dem Vertreter unterschiedlicher Positionen und Interessen unter einer neutralen Moderation zusammenfinden, um verschiedene Verbesserungsvorschläge der Mitarbeiter zu bearbeiten, die im Rahmen der Workshops aufgekommen sind. In einem ersten Schritt werden konkrete verhältnispräventive Maßnahmen erarbeitet (sog. Runde Tische 1. Ordnung), die dann in einem zweiten Schritt den Entscheidungsträgern des jeweiligen Standorts (u.a. Vorstand) als eine Art Manifest vorgestellt werden (sog. Runde Tische 2. Ordnung). TeilnehmerInnen des Runden Tisches werden einzelne TeilnehmerInnen der Workshops sein. Die Dauer pro Runder Tisch beträgt 3-4 Stunden.

Darüber hinaus wird eine Prozessevaluation Teil dieser Studie sein. Zur Vorbereitung der Leitfadenentwicklung werden bis zu vier Experteninterviews mit nicht in der Studie involvierten Personen durchgeführt. An jedem Studienort werden drei Fokusgruppen (Prä und Post-Intervention, 12 Fokusgruppen über alle Standorte) mit jeweils max. 8 Teilnehmern der Interventionsgruppe durchgeführt, um die Einstellungen, Wahrnehmungen und Erfahrungen der Teilnehmer bezüglich der Intervention zu identifizieren. Es wird ein längsschnittliches Design angewandt, d.h. dieselben Teilnehmer werden zweimal zur Teilnahme eingeladen. Sollten sich im Verlauf der Fokusgruppeninterviews noch zusätzliche Fragestellungen herauskristallisieren, werden standortübergreifend weitere webbasierte Fokusgruppen durchgeführt. Weiterhin wird im Rahmen des RCT von den TeilnehmerInnen der Interventionsgruppe bei Abschluss der Intervention mit einer Fidelity-Skala die Durchführungsadhärenz der komplexen Intervention erfasst. Schließlich werden anhand des Gesamtdatensatzes Wirkmechanismen und Moderatoren des Effekts identifiziert.

Auffrischungs-Sitzungen

Auf alle Workshops (außer dem Workshop zur Sensibilisierung der oberen Führungskräfte) folgt eine zweistündige Auffrischungs-(Booster)-Sitzung, um die Trainingseffekte zu verbessern. In diesen Booster-Sitzungen können sich Interessierte mit Trainern treffen, um Erfahrungen aus dem Training in den Klinikalltag zu übertragen. Booster-Sitzungen werden nach Abschluss aller Seminare angeboten. Sie sind offen für Teilnehmer aus den Workshops, die sich zum gleichen Thema getroffen haben.

Studiendesign

Die Studie ist als multizentrische clusterrandomisierte offene Studie mit einer Wartelistenkontrollgruppe konzipiert.

Randomisierung und Verblindung

Die Clusterrandomisierung wird vor der Rekrutierung der ersten Teilnehmer durchgeführt. Die Zuordnung erfolgt 1:1 stratifiziert durch die drei Standorte mittels einer Randomisierungsliste, die vom Institut für Medizinische Biometrie und Informatik (IMBI) erstellt wird. Aufgrund der Art der Interventionsblöcke ist eine Verblindung von Studienteilnehmern und Trainern nicht möglich.

Studiendauer

Die Gesamtdauer der Studie beträgt 12 Monate und besteht aus einer 10-monatigen Interventionsphase. Die verschiedenen Interventionsmodule unterscheiden sich in ihrer Dauer: Das erste Interventionsmodul - *Gestaltungskompetenz für gesunde Arbeit – lohnende, realisierbare Ansatzpunkte* - hat eine Dauer von sechs Zeitstunden, das zweite Interventionsmodul - *Dilemmakompetenz – schwierige Entscheidungen schaffen, ohne von ihnen geschafft zu werden* – hat eine Dauer von 12 Zeitstunden und wird über einen Zeitraum von zwei Monaten angeboten. Die *stresspräventive Führungskompetenz – teamorientierte Führung im Krankenhaus* hat eine Gesamtdauer von 12 Zeitstunden an drei Terminen. Das Interventionsmodul *Vereinbarkeit von Beruf und Familie* hat eine Dauer von einem Arbeitstag, während *Gesund Bleiben bei der Arbeit* mit einer Dauer von 10 Zeitstunden über einen Zeitraum von einem Monat angeboten wird.

Anzahl der Studienteilnehmer:

Alle Mitarbeiter der drei teilnehmenden Klinikstandorte (Aalen und Ellwangen, Heidelberg, Duisburg) sind potenzielle Teilnehmer. Pro Standort sind mindestens sechs Cluster mit insgesamt rund 720 potenziellen Teilnehmern geplant (360 Teilnehmer in der Interventions- und 360 Teilnehmer in der Wartelistenkontrollgruppe).

Die Einschlusskriterien für die Studienorte sind:

1. Bereitschaft zur Teilnahme an der Studie unabhängig von der Zuteilung zu Interventions- oder Wartelistenkontrollarm.
2. Bereitschaft zum Ausfüllen von drei Fragebögen (unabhängig von einer Teilnahme an den Workshops)

Für Mitarbeiter, die an einer oder mehreren Interventionen teilnehmen möchten, wurden folgende Einschlusskriterien formuliert:

1. Alter: 16-65 Jahre alt,

2. Schriftliche Einwilligungserklärung nach Aufklärung,

3. Ausreichende Deutschkenntnisse zum Ausfüllen der Fragebögen

4. Für das Führungskräftetraining (i) zwecks Sensibilisierung für betriebliches Gesundheitsmanagement: Position im Top-Management oder Stellvertreter. (ii) Für das Führungskräftetraining zur stresspräventiven Führungskompetenz: alle Berufsgruppen mit Management-/Führungsposition mit Ausnahme des Top-Managements,

5. Für Dilemma-Kompetenztraining: alle Berufsgruppen im Krankenhaus, die mit einer Dilemmasituation konfrontiert sind (unabhängig von der beruflichen Position),

6. Für die Vereinbarkeit von Beruf und Familie: alle Berufsgruppen im Krankenhaus mit Kindern,

7. Für Gesund Bleiben im Beruf: alle Berufsgruppen im Krankenhaus

Rekrutierung

An drei Studienstandorten (Aalen und Ellwangen, Heidelberg, Duisburg) werden geeignete Cluster identifiziert. Interessierte Mitarbeiter in diesen Clustern werden über die Studie und die verschiedenen Maßnahmen durch Informationsveranstaltungen oder durch das betriebliche Gesundheitsmanagement des jeweiligen Standorts informiert. Alle an den Interventionen interessierten Mitarbeiter werden mündlich und schriftlich informiert. Das Studienpersonal wird potenzielle Teilnehmer kontaktieren und eine schriftliche Einwilligung einholen. Für die Teilnehmer der Interventionsgruppen-Cluster wird eine Auswahl von fünf Interventionen mit den oben genannten Einschlusskriterien angeboten. Für die Teilnehmer der Wartelistenkontrollcluster werden die fünf Interventionen nach Ablauf der Studienzeit angeboten.

Alle personenbezogenen Daten werden pseudonymisiert erfasst, übertragen und gespeichert.

Während der Durchführung der Studie ist die Datenbank nur für den Datenverwalter und das Dateneingabepersonal zugänglich. Nach der Schließung der Datenbank werden auch den verantwortlichen Biometrikern Zugriffsrechte erteilt.

Die Daten werden nach den entsprechenden im IMBI gültigen Standard Operating Procedures (SOPs) verwaltet und analysiert.

Die Geschäftsdaten werden an den Lehrstuhl für Betriebswirtschaftslehre, insbesondere Organisation und Personalwesen der Heinrich-Heine-Universität Düsseldorf zur Analyse übermittelt.

Sämtliche Ergebnisse werden lediglich in anonymisierter sowie aggregierter Form der Leitungsebene des entsprechenden Krankenhauses mitgeteilt.

**5.** Ausführlichen Studienplan (mit detaillierten Angaben zur Fallzahlberechnung /-abschätzung und den statistischen Auswertemethoden/-strategien) beifügen.

s. Studienprotokoll anbei.

**6.** Begründung für die Versuche an Menschen.

Die Beantwortung unserer Forschungsfrage ist nur durch Studien an und mit im Krankenhaus Tätigen möglich.

Zur Bedeutung des Themas und der zu erwartenden Ergebnisse: siehe Studienprotokoll Punkte 4 und 13.2

Zur Auswahl der Studienteilnehmer: siehe Studienprotokoll Punkt 9

**7.** Darstellung bisher dazu vorliegender aussagekräftiger Tierversuche

Tierversuche werden nicht erfolgen.

Zur Begründung der Versuche am Menschen siehe Studienprotokoll Punkt 4

**8.** Darstellung bisher zum Projekt bzw. zur Fragestellung vorliegender Untersuchungen am Menschen. Angabe der bisherigen Erfahrungen über die verwendeten Techniken beim Menschen.

Gesundheit, Entstehung und Verlauf von Krankheiten sind durch sehr verschiedene Faktoren bestimmt. Insbesondere das berufliche Umfeld formt eine Vielzahl dieser Faktoren sowohl im positiven Sinne zum Beispiel durch die Chance, Anerkennung zu erfahren, kreativ und produktiv zu sein, soziale Kontakte zu knüpfen und als Ressource zur Bewältigung belastender Situationen zu verwenden. Aber auch im negativen Sinne, wenn die aus umfangreichen Anforderungen bestehende, modern verdichtete Arbeitswelt zum Beispiel Gefühle von Überforderung oder Entfremdung erzeugt.

Gerade in den Arbeitsbereichen der Krankenhäuser und Klinika, die in den letzten Dekaden einem enormen Wandel insbesondere bei den Arbeitsbedingungen zum Beispiel durch Kostendämpfungsmaßnahmen vollzogen haben, fehlt jedoch ein systematisiertes betriebliches Gesundheitsmanagement, welches die verschiedenen Faktoren günstig beeinflussen kann.

Krankenhausmitarbeiter sind aufgrund ihres hohen beruflichen Stressniveaus eine besonders gefährdete Gruppe (Dollard et al. 2007). In einem systematischen Review (Dollard et al. 2007) wurden die individuellen und organisatorischen Auswirkungen von arbeitsbedingtem Stress im australischen und internationalen Gesundheits- und Sozialwesen untersucht. Die Ergebnisse zeigen einen hohen beruflichen Stress im Sektor des Gesundheits- und Sozialwesens. Arbeitsstress wirkte sich sowohl auf den Einzelnen (z.B. psychische Gesundheit) als auch auf die Organisation (z.B. Fehlzeiten, Unzufriedenheit am Arbeitsplatz) negativ aus.

Obwohl die Beschäftigten im Gesundheitswesen einen erhöhten arbeitsbedingte Stress und Suizidraten aufweisen (Agerbo et al. 2007), fehlt den meisten Krankenhäusern in Deutschland ein systematisches betriebliches Gesundheitsmanagement, das Verhaltens- und Verhältnisprävention miteinander verknüpft. Ursache sei die hohe Komplexität im Krankenhaus. Als Reaktion findet häufig nur eine betriebliche Gesundheitsförderung in „kampagnefähigen“ Teilbereichen oder einzelnen Berufsgruppen statt (van Wyk und Pillay-Van Wyk 2010). Die Evidenz einzelner Interventionstypen wurde nur partiell nachgewiesen. Insgesamt mangelt es an wissenschaftlich exzellenten Studien, in denen verhaltenspräventive und verhältnispräventive Ansätzen kombiniert werden (Ruotsalainen et al. 2015). In anderen Branchen weisen einige Studien auf eine erhöhte Wirksamkeit der Kombination verhaltens- und verhältnispräventiver Interventionen hin (Tetrick und Winslow 2015). Reviews betonen den Bedarf an methodisch hochwertigen Studien im Bereich Gesundheitsförderung in Krankenhäusern (Ruotsalainen et al. 2015; van Wyk und Pillay-Van Wyk 2010).

**9.** Risiko der Komplikationen sowie Maßnahmen zur Verhütung bzw. Verminderung der Komplikationen.

Es besteht ein minimales Risiko, dass TeilnehmerInnen an der komplexen Intervention durch die Beschäftigung mit dem Thema Arbeitsstress belastet werden. Andere Teilnehmer könnten durch das Ausfüllen der Fragebögen belastet werden.

Um diese Risiken möglichst gering zu halten:

- werden alle Teilnehmer vorab über Inhalt und Form der Studie und Intervention informiert
- können Studienteilnehmer das Studienpersonal jederzeit Fragen zu Unklarheiten stellen
- sind alle Teilnehmer informiert, dass sie die Studienteilnahme jederzeit ohne Angabe von Gründen beenden können

siehe auch Studienprotokoll anbei, Punkte 11 und 14.

**10.** Darstellung der ärztlichen Beziehungen zwischen Untersucher und Patient

Es gibt zwischen den Studienteilnehmern einerseits und den Untersuchern andererseits keine ärztlichen Beziehungen.

**11.** Ist der Patient über die Diagnose bzw. die Art der Erkrankung informiert?

Bei der komplexen Intervention handelt es sich um einen verhaltens- und verhältnispräventiven Ansatz, der nicht zum Ziel hat, bereits bestehende psychische Erkrankungen zu thematisieren. Zielgruppe sind belastete Krankenhausmitarbeiter. Weitere Diagnostik findet im Rahmen der Studie nicht statt. Aufklärungsbogen und Einwilligungserklärungen sind diesem Antrag beigefügt.

**12.** Darstellung, wie die Versuchsperson über das Untersuchungsprogramm, die Notwendigkeit und die Risiken informiert wurde und unter welchen Bedingungen die Zustimmung zum Versuch gegeben wurde.

Wir werden mit Flyern und Booklets auf unsere Studie aufmerksam machen. Zusätzlich werden potentielle Studienteilnehmer im Rahmen von Pflichtweiterbildungen und Dienstbesprechungen auf unsere Studie aufmerksam gemacht. Interessierte Personen können sich dann bei uns melden. Die Studienteilnahme ist freiwillig. Es bestehen keine Abhängigkeits- oder Behandlungsverhältnisse zwischen Untersuchern und möglichen Studienteilnehmern (s. auch Punkt 10 dieses Antrags oben). Alle Studienteilnehmer erhalten Aufklärungsbögen (als Anlage anbei), und vor Studieneinschluss ist die schriftliche Einwilligung der Studienteilnehmer notwendig (ebenfalls als Anlage anbei).

**13.** Falls nur eine mündliche Zustimmung gegeben wurde, muss begründet werden, warum die schriftliche Einwilligung nicht eingeholt werden konnte.

Es erfolgt eine schriftliche Zustimmung der Studienteilnehmer.

**14.** Information der auf der Station tätigen Ärzte über die Untersuchung an den von ihnen betreuten Patienten.

Wir werden die auf Station tätigen Ärzte über unsere Studie informieren. Eine Studienteilnahme ihrer Patienten ist nicht vorgesehen und wird daher nicht erfolgen.

**15.** Verantwortung für die klinische Kontrolle der Patienten

Es erfolgt keine regelmäßig klinische Kontrolle der Studienteilnehmer im Rahmen unserer Studie.

**16.** Wird das Forschungsprojekt auf dem Krankenblatt oder auf der Fieberkurve kenntlich gemacht?

Nein, es wird nicht kenntlich gemacht werden.

**17.** Art der klinischen Untersuchungen bei gesunden Kontrollpersonen.

Es werden gesunde Personen an der komplexen Intervention teilnehmen, allerdings sind keine klinischen Untersuchungen vorgesehen.

**18.** Art der Entschädigung der Versuchsperson.

Die Teilnahme an der komplexen Intervention und an den Fokusgruppeninterviews zählen für die Studienteilnehmer als Arbeitszeit. In den qualitativen Fokusgruppeninterviews erhalten die Teilnehmer als Aufwandsentschädigung für die Teilnahme in einer Fokusgruppe einen Büchergutschein im Wert von 15 Euro. Die Interviewpartner der Experteninterviews erhalten ebenfalls einen solchen Gutschein. Das Ausfüllen der Fragebögen gilt nicht als Arbeitszeit.

**19.** Art und Höhe der Versicherung für Versuchspersonen, Projektleiter und Mitarbeiter.

 Es handelt sich um ein Projekt, welches unter Verantwortung des

Abteilungsleiters durchgeführt werden soll, so dass die allgemeinen

Haftungsgrundsätze gelten.

 Für Personenschäden besteht eine zusätzliche Haftpflichtversicherung mit einer Deckungssumme von Euro ……………….. bei: …………………..

 Für Personenschäden besteht eine Wegeunfallversicherung mit einer Deckungssumme von Euro ……………….. bei: …………………..

**20.** Es handelt sich um eine Studie mit einem industriellen Auftraggeber, bei der die für die Begutachtung durch die Ethikkommission anfallenden Kosten diesem in Rechnung gestellt werden.

 ja  nein

Anschrift der Firma: …………………………………..

…………………………………..

…………………………………..

**21.** Angaben über finanzielle Zuwendungen des industriellen Auftraggebers:

 pro vollständig ausgefülltem CRF bzw. rekrutierten Patienten:

€ ………………………….

 an den einzelnen Patienten:

€ ………………………….

 insgesamt: € ………………………….

Nicht zutreffend

**22.** Das Forschungsprogramm wurde geprüft und Zustimmung gegeben:

Datum:

Projekt- und Abteilungsleiter Ärztlicher Direktor

(Prof. Dr. Harald Gündel) (BKH Günzburg, Prof. Dr. Thomas Becker)

1. *Unterschriften* ***immer*** *durch Maschinenschrift oder Stempel kenntlich machen!*

**Literaturverzeichnis**

Agerbo, E.; Gunnell, D.; Bonde, J. P.; Mortensen, P. B.; Nordentoft, M. (2007): Suicide and occupation: the impact of socio-economic, demographic and psychiatric differences. *Psychological medicine* 37 (8), S. 1131–1140.

Dollard, M. F.; LaMontagne, A. D.; Caulfield, N.; Blewett, V.; Shaw, A. (2007): Job stress in the Australian and international health and community services sector: A review of the literature. *Int J Stress Manag* 14 (4), S. 417–445.

Mohr, G.; Rigotti, T.; Müller, A. (2005): Irritation - ein Instrument zur Erfassung psychischer Beanspruchung im Arbeitskontext. Skalen- und Itemparameter aus 15 Studien [Irritation - an instrument assessing mental strain in working contexts. Scale and item parameters from 15 studies]. *Zeitschrift für Arbeits- und Organisationspsychologie A&O* 49 (1), S. 44–48.

Ruotsalainen, J. H.; Verbeek, J. H.; Mariné, A.; Serra, C. (2015): Preventing occupational stress in healthcare workers. *The Cochrane database of systematic reviews* (4), CD002892.

Tetrick, L. E.; Winslow, C. J. (2015): Workplace Stress Management Interventions and Health Promotion. *Annu. Rev. Organ. Psychol. Organ. Behav.* 2 (1), S. 583–603.

van Wyk, B. E.; Pillay-Van Wyk, V. (2010): Preventive staff-support interventions for health workers. *The Cochrane database of systematic reviews* (3), CD003541.

1. Bitte fügen Sie hier bei multizentrischen Studien die Leiterin/den Leiter der Gesamtstudie ein. [↑](#footnote-ref-1)
