# Supplementary material for: Effectiveness of a multilevel intervention to improve mental health of hospital workers: The SEEGEN multicenter cluster randomized controlled trial
Source: PLoS One. 2025 Aug 21;20(8):e0330490. doi: 10.1371/journal.pone.0330490 (PMC12370060; doi:10.1371/journal.pone.0330490)
Supplement: S3 Document — (DOCX) [file pone.0330490.s005.docx]

**University of Ulm No. / Year**

# Ethics Committee

Presentation of research projects on scientific investigations on humans

1. Title of the project:

Mental health in the workplace hospital (SEEGEN)

Study director^[[1]](#footnote-1)^ : Prof. Dr. med. Harald Gündel

Number of patients/subjects included in the study: N = 720

1. Quantitative cluster-randomized study:

Three study centers, 18 clusters in total (i.e. 6 per study center)

- Ostalbkliniken Aalen (6 clusters with 40 participating employees) n = 240
- Heidelberg University Hospital (6 clusters with 40 participating employees)

n = 240

- Helios Klinikum Duisburg (6 clusters with 40 participating employees)

n = 240

1. Qualitative focus group interviews - process evaluation (N = 32)

x This is a prospective study.

This is a retrospective study.

x The data is processed pseudonymously.

The data is completely anonymized after completion of the study,

It is then no longer possible to assign the data to individual persons.

**2.** Project leader:

Prof. Dr. med. Harald Gündel, Medical Director of the Clinic for Psychosomatic Medicine and Psychotherapy, Ulm University Hospital

a) Medical cooperation partners / employees:

- - Prof. Dr. med. Peter Angerer, Institute for Occupational, Social and Environmental Medicine. University Hospital Düsseldorf
  - Prof. Dr. med. Stephan Zipfel, Prof. Dr. med. Monika Rieger and Dr. med. Florian Junne, Clinic for Psychosomatic Medicine, University Hospital Tübingen
  - Dr. med. Eva Rothermund, Clinic for Psychosomatic Medicine, Ulm University Hospital
  - Dr. med. Imad Maatouk, Clinic for Psychosomatic Medicine, Heidelberg University Hospital

b) Non-medical cooperation partners / employees:

- - Prof. Dr. phil. Andreas Müller and Melanie Genrich, Industrial and Organizational Psychology, University of Duisburg-Essen
  - Prof. Dr. rer.soc. Jochen Schweitzer-Rothers, Ulrike Bossmann, Dr. sc. hum. Julika Zwack, Marieke Born, Antonia Drews, Institute for Medical Psychology, Heidelberg University Hospital
  - Felicitas Stuber and Elena Tsarouha, Clinic for Psychosomatic Medicine, University Hospital Tübingen
  - Prof. Dr. phil. Ute Ziegenhain, Manuela Gulde and Franziska Köhler-Dauner, Department of Child and Adolescent Psychiatry, Ulm University Hospital
  - Dr. biol. hum. Lucia Jerg-Bretzke, Department of Psychosomatic Medicine, Section of Medical Psychology, Ulm University Hospital
  - Dr. biol. hum Nadine Mulfinger and Dr. Marc N. Jarczok, Department of Psychosomatic Medicine, Ulm University Hospital
  - Madeleine Helaß and Sebastian Götz, Department of Psychosomatic Medicine, Heidelberg University Hospital
  - PD Dr. Bernd Puschner, Maja Stiawa, Martin Peters, Department of Psychiatry and Psychotherapy II, University of Ulm
  - Prof. Dr. sc. hum. Meinhard Kieser, Dr. sc. hum. Anja Sander, Christina Klose, Regina Brinster and Ronald Limprecht, Institute of Medical Biometry and Informatics, Department of Medical Biometry, University of Heidelberg
  - Prof. Dr. rer. pol. Stefan Süß, Dr. rer. oec. Sascha Ruhle and Elena Gesang, Chair of Business Administration, in particular Organization and Human Resources, Heinrich Heine University Düsseldorf
  - Dr. PH Karl Blum, Head of Research Division, Board of Directors, Deutsches Krankenhausinstitut e.V.
  - Britta Worringer, Institute for Occupational, Social and Environmental Medicine, Düsseldorf University Hospital

**3.** Location of the intended investigations:

- Ostalbkliniken Aalen, Ellwangen and Aalen sites,
- Heidelberg University Hospital,
- Helios Clinic, Duisburg site

**4.** Description of the research program:

The research project investigates whether a complex intervention consisting of behavioral and relational preventive elements leads to an improvement in the subjectively perceived emotional and cognitive stress of hospital employees, measured with the irritation scale (Mohr et al. 2005) compared to standard care. The complex intervention will be implemented at three hospital sites and evaluated in a cluster randomized trial.

The study will also assess the effects of the complex intervention on subjective psychological well-being, psychosocial safety climate, perceived job performance as well as perceived job rewards, health-related quality of life, perception of working conditions, emotion regulation strategies, social support at work, leadership and communication style, work-family balance and organizational indicators at individual and hospital/clinic level.

The main outcome will be the change in the mean score of the irritation scale (Mohr et al. 2005) from baseline (T0) to 11 months after baseline (T2*). The irritation scale (eight items) assesses the emotional and cognitive stress in the work environment. The items are measured on a 7- point Likert scale ranging from 1 (not at all) to 7 (almost completely correct).

All participants in a cluster who decide not to take part in a workshop are asked to complete an abbreviated questionnaire (see appendix) at three measurement points. In this way, the effects of the ratio-preventive intervention approach can be examined.

The complex intervention was developed on the basis of a previous two-year pilot phase in which the individual interventions were tested and evaluated. The individual intervention modules are described in more detail below

1. The workshop - *Design competence for healthy work - worthwhile, feasible starting points* - which lasts six hours, focuses on promoting the competence to design healthy and motivating working conditions in the team and developing structures for cross-departmental work on organizational improvement measures. The awareness-raising workshop is aimed at senior nursing staff and senior doctors who want to systematically reduce organizational stressors for the team. In the workshop, managers learn about the significant influence that working conditions have on the health and performance of their employees, the four key steps that need to be considered when it comes to healthy work design, and how they can develop and implement specific organizational improvement measures at their clinic on an interprofessional and cross-departmental basis.
2. The *dilemma competence* workshop *- making difficult decisions without being made by them* - is aimed at employees from all professional groups who frequently have to make difficult decisions and would like to improve their handling of such decision-making situations. The training promotes decision-making skills in situations in which there is no one hundred percent coherent solution, but every option is associated with disadvantages. Among other things, the training conveys an awareness of one's own role in the hospital organization, including the often contradictory requirements and conflicting goals between different departments, an awareness of one's own decision-making rules ("I must always make the right decision") and the organizational culture. The two-day dilemma skills training takes place over two days, four to eight weeks apart, in groups of 10 to 20 participants.
3. *The workshop stress-preventive leadership skills: strengthening resources through team-oriented leadership in hospitals - is* aimed at middle management (e.g. senior physicians, nursing department managers, functional/team managers from all professional groups). The seminar offers the opportunity to get to know suggestions for dealing with stress and pressure situations, to expand stress-preventive and employee-oriented leadership skills using innovative leadership concepts, to further develop one's own skills in the area of communication and interaction in order to strengthen the resource "team" in complex relationships. The seminar with a maximum of 20 participants takes place on two days, approximately three weeks apart.
4. The "Balancing family and career" workshop, which lasts six hours with a maximum of 15 participants, was specially developed for employees in the nursing and medical services who are in the family phase. The aim is to help them cope better with stressful situations at the interface between work and family life. Participants in the workshop are made aware of the need to expand their individual scope of action and to recognize, analyse and defuse potential stress situations. This includes, among other things Analyzing their own family and professional situation in order to identify obstacles and resources for a successful work-life balance, communicating facts about the connection between stress experience, stress reaction and the influence of personal stress on the relationship with the child as well as developmental psychological findings for practical everyday parenting, learning practical exercises and relief strategies (breathing and yoga exercises) that can be easily integrated into everyday life.
5. *Staying healthy at work - learning strategies for self-care and resource activation*: This group intervention with up to 15 participants from all professional groups covers various approaches to coping with age- and stress-related changes with instructions on how to implement them in everyday life on two dates approximately 4 weeks apart. The training includes joint reflection on stress and age-related changes, sharing experiences and personal experiences in the workplace as well as teaching and practicing methods to improve the perception of signs of stress

In addition to the workshops, round tables are held to facilitate organizational change and develop concrete proposals for action. One round table will be held for each type of workshop (with the exception of the workshop *"Design competence for healthy work - worthwhile, feasible starting points*"), i.e. four round tables will be held at each location for the intervention and control groups. The round tables are a participatory instrument at which representatives of different positions and interests come together under neutral moderation to work on various suggestions for improvement made by employees during the workshops. In a first step, concrete measures to prevent the situation are developed (so-called first-order round tables), which are then presented to the decision-makers at the respective location (including the Management Board) in a second step as a kind of manifesto (so-called second-order round tables). The participants of the round table will be individual participants of the workshops. The duration of each round table is 3-4 hours.

In addition, a process evaluation will be part of this study. In preparation for the development of the guidelines, up to four expert interviews will be conducted with people not involved in the study. Three focus groups (pre- and post-intervention, 12 focus groups across all sites) will be conducted at each study site, each with a maximum of 8 participants from the intervention group, in order to identify the attitudes, perceptions and experiences of the participants regarding the intervention. A longitudinal design will be used, i.e. the same participants will be invited to participate twice. If additional questions emerge in the course of the focus group interviews, further web-based focus groups will be conducted across all sites. Furthermore, as part of the RCT, the adherence to the complex intervention will be assessed by the participants in the intervention group at the end of the intervention using a fidelity scale. Finally, the overall data set will be used to identify mechanisms of action and moderators of the effect.

Refresher sessions

All workshops (except the workshop to sensitize senior managers) are followed by a two-hour refresher (booster) session to improve the training effects. In these booster sessions, interested parties can meet with trainers to transfer experiences from the training to everyday clinical practice. Booster sessions are offered after completion of all seminars. They are open to participants from the workshops who have met on the same topic.

Study design

The study is designed as a multicenter cluster randomized open trial with a waiting list control group.

Randomization and blinding

Cluster randomization will be performed prior to recruitment of the first participants. The allocation will be stratified 1:1 by the three sites using a randomization list created by the Institute of Medical Biometry and Informatics (IMBI). Due to the nature of the intervention blocks, blinding of study participants and trainers is not possible.

Duration of the study

The total duration of the study is 12 months and consists of a 10-month intervention phase. The various intervention modules differ in their duration: the first intervention module - *design competence for healthy work - rewarding, feasible starting points* - has a duration of six hours, the second intervention module - *dilemma competence - making difficult decisions without being overcome by them* - has a duration of 12 hours and is offered over a period of two months. The *stress-preventive leadership competence - team-oriented leadership in hospitals* has a total duration of 12 hours over three sessions. The *work-life balance* intervention module has a duration of one working day, while *staying healthy at work* is offered over a period of one month with a duration of 10 hours.

Number of study participants:

All employees at the three participating hospital sites (Aalen and Ellwangen, Heidelberg, Duisburg) are potential participants. At least six clusters with a total of around 720 potential participants are planned per site (360 participants in the intervention group and 360 participants in the waiting list control group).

The inclusion criteria for the study sites are:

1. Willingness to participate in the study regardless of allocation to intervention or waiting list control arm.
2. Willingness to complete three questionnaires (regardless of participation in the workshops)

The following inclusion criteria were formulated for employees wishing to participate in one or more interventions:

1. Age: 16-65 years old,

2. Written informed consent,

3. Sufficient knowledge of German to complete the questionnaires

4. For management training (i) to raise awareness of occupational health management: position in top management or deputy. (ii) For management training on stress-preventive leadership skills: all professional groups with a management/leadership position with the exception of top management,

5. For dilemma competence training: all professional groups in the hospital who are confronted with a dilemma situation (regardless of professional position),

6. For work-life balance: all professional groups in the hospital with children,

7. For staying healthy at work: all professional groups in the hospital

Recruitment

Suitable clusters are identified at three study locations (Aalen and Ellwangen, Heidelberg, Duisburg). Interested employees in these clusters are informed about the study and the various measures through information events or through the company health management of the respective location. All employees interested in the interventions will be informed verbally and in writing. The study staff will contact potential participants and obtain written consent. Participants in the intervention group clusters will be offered a choice of five interventions with the above inclusion criteria. For participants in the waiting list control clusters, the five interventions will be offered at the end of the study period.

All personal data will be collected, transferred and stored pseudonymously.

During the conduct of the study, the database is only accessible to the data administrator and the data entry personnel. After the database is closed, access rights are also granted to the biometricians responsible.

The data are managed and analyzed according to the corresponding Standard Operating Procedures (SOPs) valid at IMBI.

The business data is sent to the Chair of Business Administration, in particular Organization and Human Resources at Heinrich Heine University Düsseldorf for analysis.

All results are only communicated in anonymized and aggregated form to the management level of the respective hospital.

**5.** Attach a detailed study plan (with detailed information on the calculation/estimation of case numbers and the statistical evaluation methods/strategies).

s. Study protocol attached.

**6.** Justification for the human trials.

The answer to our research question is only possible through studies on and with people working in hospitals.

On the importance of the topic and the expected results: see study protocol points 4 and 13.2

On the selection of study participants: see study protocol point 9

**7.** Presentation of meaningful animal experiments available to date

Animal experiments will not be conducted.

For justification of the experiments on humans, see study protocol point 4

**8.** Description of existing human studies on the project or the research question. Details of previous experience with the techniques used in humans.

Health, development and progression of diseases are determined by very different factors. The professional environment in particular shapes a large number of these factors, both in a positive sense, for example through the opportunity to experience recognition, to be creative and productive, to establish social contacts and to use them as a resource for coping with stressful situations. But also in a negative sense, for example, when the modern, condensed working environment consisting of extensive demands creates feelings of excessive demands or alienation.

However, especially in the working areas of hospitals and clinics, which have undergone enormous change in recent decades, particularly in terms of working conditions, for example due to cost-cutting measures, there is a lack of systematic occupational health management that can have a positive influence on the various factors.

Hospital employees are a particularly vulnerable group due to their high level of occupational stress (Dollard et al. 2007) . A systematic review (Dollard et al. 2007) examined the individual and organizational effects of work-related stress in the Australian and international health and social care sector. The results show a high level of occupational stress in the health and social care sector. Work stress had a negative impact on both the individual (e.g. mental health) and the organization (e.g. absenteeism, job dissatisfaction).

Although employees in the healthcare sector have increased work-related stress and suicide rates (Agerbo et al. 2007) , most hospitals in Germany lack a systematic occupational health management system that combines behavioral and situational prevention. The reason for this is the high level of complexity in hospitals. In response, workplace health promotion often only takes place in "campaignable" sub-areas or individual occupational groups (van Wyk and Pillay-Van Wyk 2010) . The evidence for individual types of intervention has only been partially proven. Overall, there is a lack of scientifically excellent studies in which behavioral prevention and relationship prevention approaches are combined (Ruotsalainen et al. 2015) . In other sectors, some studies point to the increased effectiveness of combining behavioral and behavioral prevention interventions (Tetrick and Winslow 2015) . Reviews emphasize the need for methodologically high-quality studies in the field of health promotion in hospitals (Ruotsalainen et al. 2015; van Wyk and Pillay-Van Wyk 2010) .

**9.** Risk of complications and measures to prevent or reduce complications.

There is a minimal risk that participants in the complex intervention will be burdened by dealing with the topic of work stress. Other participants could be burdened by completing the questionnaires.

To keep these risks as low as possible

- all participants are informed in advance about the content and form of the study and intervention
- study participants can ask the study staff questions about anything unclear at any time
- all participants are informed that they can end their participation in the study at any time without giving reasons

see also study protocol attached, points 11 and 14.

**10.** Description of the medical relationship between investigator and patient

There are no medical relationships between the study participants on the one hand and the investigators on the other.

**11.** Is the patient informed about the diagnosis or the nature of the disease?

The complex intervention is a behavioral and relational prevention approach that does not aim to address pre-existing mental illness. The target group is stressed hospital employees. No further diagnostics are carried out as part of the study. The information sheet and consent forms are attached to this application.

**12.** Description of how the subject was informed about the study program, the necessity and the risks and under which conditions consent to the study was given.

We will draw attention to our study with flyers and booklets. In addition, potential study participants will be made aware of our study as part of mandatory further training and service meetings. Interested persons can then contact us. Participation in the study is voluntary. There are no dependency or treatment relationships between investigators and potential study participants (see also point 10 of this application above). All study participants will receive information sheets (attached), and written informed consent is required from the study participants prior to study inclusion (also attached).

**13.** If only verbal consent was given, reasons must be given as to why written consent could not be obtained.

Written consent will be obtained from the study participants.

**14.** Informing the doctors working on the ward about the examination of the patients they patients under their care.

We will inform the doctors working on the ward about our study. Participation of their patients in the study is not planned and will therefore not take place.

**15.** Responsibility for the clinical monitoring of patients

There will be no regular clinical monitoring of study participants as part of our study.

**16.** Will the research project be indicated on the patient's medical chart or fever curve?

No, it will not be indicated.

**17.** Type of clinical examinations in healthy control subjects.

Healthy individuals will participate in the complex intervention, but no clinical examinations are planned.

**18.** Type of compensation for the test subject.

Participation in the complex intervention and in the focus group interviews will count as working time for the study participants. In the qualitative focus group interviews, the participants receive a book voucher worth 15 euros as compensation for their participation in a focus group. The interviewees in the expert interviews also received such a voucher. Completing the questionnaires does not count as working time.

**19.** Type and amount of insurance for test subjects, project managers and employees.

 This is a project that is to be carried out under the responsibility of the

the head of department, so that the general principles of liability apply.

principles of liability apply.

 There is additional liability insurance for personal injury with a sum insured of Euro ....................: .......................

 For personal injury there is a commuting accident insurance with a sum insured of Euro .................... at: .......................

**20.** This is a study with an industrial client for which the costs incurred for the review by the ethics committee will be charged to the client.

 yes  no

Address of the company: .........................................

.........................................

.........................................

**21.** Information on financial contributions from the industrial client:

 per completed CRF or recruited patient:

€ ...............................

 to the individual patient:

€ ...............................

 in total: € ...............................

Not applicable

**22.** The research program has been reviewed and approval has been given:

Date:

Project and department head Medical Director

(Prof. Dr. Harald Gündel) (BKH Günzburg, Prof. Dr. Thomas Becker)

1. *Signatures* ***must always*** *be typed or stamped!*

**Bibliography**

Agerbo, E.; Gunnell, D.; Bonde, J. P.; Mortensen, P. B.; Nordentoft, M. (2007): Suicide and occupation: the impact of socio-economic, demographic and psychiatric differences. *Psychological medicine* 37 (8), pp. 1131-1140.

Dollard, M. F.; LaMontagne, A. D.; Caulfield, N.; Blewett, V.; Shaw, A. (2007): Job stress in the Australian and international health and community services sector: A review of the literature. *Int J Stress Manag* 14 (4), pp. 417-445.

Mohr, G.; Rigotti, T.; Müller, A. (2005): Irritation - an instrument assessing mental strain in working contexts. Scale and item parameters from 15 studies [Irritation - an instrument assessing mental strain in working contexts. Scale and item parameters from 15 studies]. *Journal of Industrial and Organizational Psychology A&O* 49 (1), pp. 44-48.

Ruotsalainen, J. H.; Verbeek, J. H.; Mariné, A.; Serra, C. (2015): Preventing occupational stress in healthcare workers. *The Cochrane database of systematic reviews* (4), CD002892.

Tetrick, L. E.; Winslow, C. J. (2015): Workplace stress management interventions and health promotion. *Annu. Rev. Organ. Psychol. Organ. Behav.* 2 (1), pp. 583-603.

van Wyk, B. E.; Pillay-Van Wyk, V. (2010): Preventive staff-support interventions for health workers. *The Cochrane database of systematic reviews* (3), CD003541.

1. In the case of multicenter studies, please enter the head of the overall study here. [↑](#footnote-ref-1)
